# Contribution of Colonic Fermentation and Fecal Water Toxicity to the Pathophysiology of Lactose-Intolerance

**DOI:** 10.3390/nu7095349

**Published:** 2015-09-08

**Authors:** Karen Windey, Els Houben, Lise Deroover, Kristin Verbeke

**Affiliations:** 1 Translational Research Center for Gastrointestinal Disorders (TARGID), KU Leuven, Leuven 3000, Belgium; E-Mails: windey.karen@gmail.com (K.W.); lise.deroover@med.kuleuven.be (L.D.); 2 Laboratory Medicine, University Hospitals Leuven, Leuven 3000, Belgium; E-Mail: els.houben@uzleuven.be; 3 Leuven Food Science and Nutrition Research Centre (LFoRCe), KU Leuven, Leuven 3000, Belgium

**Keywords:** lactose intolerance, lactose malabsorption, colonic fermentation, metabolomics, cytotoxicity

## Abstract

Whether or not abdominal symptoms occur in subjects with small intestinal lactose malabsorption might depend on differences in colonic fermentation. To evaluate this hypothesis, we collected fecal samples from subjects with lactose malabsorption with abdominal complaints (LM-IT, *n* = 11) and without abdominal complaints (LM-T, *n* = 8) and subjects with normal lactose digestion (NLD, *n* = 15). Lactose malabsorption was diagnosed using a ^13^C-lactose breath test. Colonic fermentation was characterized in fecal samples at baseline and after incubation with lactose for 3 h, 6 h and 24 h through a metabolomics approach using gas chromatography-mass spectrometry (GC-MS). Fecal water cytotoxicity was analyzed using a colorimetric assay. Fecal water cytotoxicity was not different between the three groups (Kruskall-Wallis *p* = 0.164). Cluster analysis of the metabolite patterns revealed separate clusters for NLD, LM-T and LM-IT samples at baseline and after 24 h incubation with lactose. Levels of 5-methyl-2-furancarboxaldehyde were significantly higher in LM-IT and LM-T compared to NLD whereas those of an unidentified aldehyde were significantly higher in LM-IT compared to LM-T and NLD. Incubation with lactose increased short chain fatty acid (SCFA) concentrations more in LM-IT and LM-T compared to NLD. In conclusion, fermentation patterns were clearly different in NLD, LM-IT and LM-T, but not related to differences in fecal water cytotoxicity.

## 1. Introduction

Humans are generally born with the ability to digest lactose, a disaccharide consisting of β-d-glucose and β-d-galactose, due to the presence of lactase at the brush border of the small intestine. In about 75% of the world population the activity of this enzyme decreases after weaning (primary hypolactasia or lactase-nonpersistence), resulting in incomplete digestion of lactose and lactose malabsorption in adulthood [1]. Lactose malabsorption also occurs secondary to intestinal diseases, such as celiac disease, infectious enteritis or Crohn’s disease [2]. Very rarely, lactase deficiency is congenital due to an autosomal recessive genetic disorder, preventing lactase expression from birth [3]. Whereas some people with lactose malabsorption are asymptomatic, lactose-nonpersisters often experience symptoms like abdominal pain, bloating, excess flatulence or diarrhea. Lactose intolerance refers to the onset of one or more symptoms after consumption of lactose-containing food in individuals with lactose malabsorption [4]. At present, the precise mechanism that triggers the generation of symptoms in lactose-intolerance is not known.

In a study by Vonk *et al.* (2003), lactose intolerant subjects were divided into two groups, according to the severity of their symptoms [5]. One group was characterized by diarrhea, while the other group only experienced mild symptoms without diarrhea. The degree of lactose digestion in the small intestine was not different between both groups, indicating a similar lactase activity and leading them to the hypothesis of a “colon resistance factor” [5]. It has been suggested that the symptoms experienced by lactose intolerant patients are the result of a different fermentation of lactose in the colon. When lactose is malabsorbed and enters the colon, it is fermented by the resident microbiota into a variety of metabolites including lactate, formate, succinate and the SCFA, acetate, propionate, butyrate as well as gases including hydrogen (H_2_), carbon dioxide (CO_2_) and methane (CH_4_) [6]. When incubating fecal samples from lactose-tolerant and -intolerant subjects with lactose, the samples from the lactose-intolerant subjects showed faster production rates of d- and l-lactate, acetate, propionate and butyrate, indicating a difference in the colonic fermentation with healthy subjects [7]. These fermentation metabolites could be responsible for abdominal pain, excessive flatulence and bloating. Possible mechanisms are an increase in the osmotic load, changes in colonic motility and an increased colonic sensitivity caused by the accumulation of SCFA in the colon [8,9,10]. However, these last two mechanisms have only been observed in rats. Campbell *et al.* introduced the bacterial metabolic toxin hypothesis, stating that also other bacterial metabolites, such as alcohols, aldehydes, acids and ketones, resulting from carbohydrate fermentation may play a role in the pathogenesis of lactose-intolerance. These metabolites might inhibit bacterial growth and affect eukaryotic cells [11]. In our previous studies in which we associated metabolite profiles of fecal samples to fecal water cytotoxicity, we found that propionic acid, medium chain fatty acids, 1-octanol and heptanal were more prevalent in the samples that exhibited the highest cytotoxicity (10), supporting the toxic effect of these compounds. Also the latest report of the Rome Consensus Conference suggests that measurement of volatile organic compounds should be encouraged to add new insights on intestinal pathophysiology [12].

We hypothesize that the fermentation pattern is different in healthy controls compared to subjects with lactose-malabsorption without symptoms (lactose tolerant subjects) and subjects with symptoms (lactose-intolerant subjects). Production of different metabolites might explain toxicity and the generation of symptoms.

## 2. Experimental Section

### 2.1. Study Population and Study Design

Fecal samples were collected from healthy subjects with normal lactose digestion (NLD) and subjects with low lactose digestion and lactose malabsorption (LM). The group of subjects with LM was divided into a group that presented with abdominal complaints during the ^13^C-lactose breath test (LM with lactose intolerance, LM-IT) and a group with no complaints (LM with lactose tolerance, LM-T) during the ^13^C-lactose breath test. All participants had a regular dietary pattern, were older than 18 years and had a body mass index (BMI) between 18.5 and 27 kg/m^2^. None of the participants were vegetarians or on a specific diet, underwent abdominal surgery (except for appendectomy) prior to the study nor had a history of chronic gastro-intestinal conditions such as inflammatory bowel disease, irritable bowel syndrome or celiac disease. None of the participants ingested anti-, pre- or probiotics one month prior to the study. Finally, all participants were free of medication affecting the gut function two weeks prior to the study.

Patients with LM were selected from a database from the University Hospitals Leuven. Selection criteria were a positive ^13^C-lactose breath test (performed between January 2013 and December 2014), a clear description of the presence or absence of complaints experienced during the breath test to allow classification as LM-IT or LM-T and living in the proximity of Leuven to assure a rapid transport of the collected samples to the laboratory. Selected patients were sent a letter to inform them about the study and to ask them to participate. Patients that agreed to participate filled in a questionnaire on their demographic characteristics, medical history, dietary habits and drug use to check inclusion and exclusion criteria. Subjects with presumed normal lactose digestion were recruited amongst colleagues at the KU Leuven. They performed a ^13^C-lactose breath test to confirm normal lactose digestion and also completed the questionnaire. Eligible subjects (NLD, LM-IT and LM-T) collected a fecal sample under anaerobic conditions.

This study was conducted according to the guidelines laid down in the Declaration of Helsinki and was approved by the Ethics Committee of the University Hospitals Leuven. The trial was registered at ClinicalTrial.gov (clinical trial number: NCT02171403). All subjects gave their written informed consent before participation.

### 2.2. Handling of the Fecal Samples

Fecal samples were collected by the participants in dedicated recipients containing an Anaerocult strip (Merck, Darmstadt, Germany) to create anaerobic conditions during transport. Within 24 h after collection of the sample, an aliquot of the fecal sample was ultracentrifuged (2 h, 4 °C, 50,000 *g*) to prepare fecal water that was stored at −20 °C until further analysis. Another aliquot was used immediately after arrival at the laboratory to prepare 2.5% m/V fecal slurries in oxygen-free phosphate-buffered saline. One fraction of the slurry was analyzed immediately to evaluate the baseline fecal metabolite pattern. The remaining fractions were incubated for 3 h, 6 h and 24 h at 37 °C with 6.25 mg lactose or without lactose (control).

### 2.3. Analytical Methods

#### 2.3.1. Evaluation of the Ability to Digest and Absorb Lactose: ^13^C-lactose Breath Test

After an overnight fast, subjects collected two baseline breath samples in Exetainers^®^ (Labco Ltd, Ceredigion, UK), and ingested 50 g of ^13^C-labelled lactose (naturally enriched, AP 1.097), dissolved in 250 mL of water. Afterwards they rinsed the glass with an additional 250 mL of water. Consequently, breath samples were collected in duplicate with 30-min intervals up to 4 h after ingestion of the ^13^C-lactose. Subjects were also asked to report symptoms or discomfort observed during the test day.

H_2_-levels were measured in one set of samples using a gas chromatograph (GC, Trace GC Ultra, Interscience, Louvain-la-Neuve, Belgium) coupled to a thermal conductivity detector (TCD). The GC was equipped with a packed precolumn (Hayesep-N; 0.25 m; 80–100 mesh; 1/8”SS, Restek, Bellefonte, PA, USA) followed by a packed column (Carboxen 1000; 1.5 m; 60–80 mesh; 1/8” SS, Restek, Bellefonte, PA, USA) and used nitrogen 5.0 as a carrier gas with a constant pressure of 96 kPa. Results were expressed in ppm. An increase in H_2_-excretion with 20 ppm above baseline was considered as an indication of bacterial metabolism of lactose. In the second set of samples, ^13^CO_2_-levels were measured using isotope-ratio mass spectrometry (IRMS) (ABCA, Sercon, Crewe, England) and converted to percentage of administered dose. Normal lactose digestion was defined as a cumulative ^13^CO_2_-excretion after 4 h exceeding 14.5% of administered dose and the absence of increased H_2_-excretion [13].

#### 2.3.2. Analysis of Fecal Metabolites Using GC-MS

Volatile organic compounds were analyzed using a GC-MS (Trace GC Thermoquest, Rodano, Italy and Tempus II, Thermo Electron, San Jose, CA, USA) coupled online to a purge and trap sample preparation system as previously described [14]. Relative indices *versus* 2-ethylbutyrate as internal standard were calculated for all compounds. A number of metabolites, selected as markers for saccharolytic fermentation (acetate, propionate and butyrate) and proteolytic fermentation (isobutyrate and isovalerate, dimethylsulfide and p-cresol) were also absolutely quantified. SCFA were determined as the sum of acetate, propionate and butyrate and branched chain fatty acids (BCFA) as the sum of isobutyrate and isovalerate. SCFA and BCFA were quantified using 2-ethylbutyrate as internal standard, whereas *p*-cresol was quantified *versus* 2,6-dimethylphenol and dimethylsulfide *versus* diethylsulfide.

#### 2.3.3. Analysis of Fecal Water Cytotoxicity: WST-1 Assay

Cytotoxicity of fecal water was measured on HT-29 cells using a colorimetric cell viability assay based on the cleavage of a tetrazolium salt in the mitochondria of living cells to produce a colored formazan derivative, as previously described [15]. Briefly, after growing for 24 h, HT-29 cells were exposed to serial dilutions of fecal water samples (1/4–1/1024) for 72 h. Cell viability was measured by adding 10 µL of the tetrazolium salt, 4-[3-[4-Iodophenyl]-2-4-(4-nitrophenyl)-2H-5-tetrazolio-1,3-benzene disulfonate (WST-1, Roche Diagnostics, Basel, Switzerland) to the cells. Triton X-100 (0.5%) was used as positive control and medium as negative control. After 4 h UV-absorbance (abs) at 450 nm was measured spectrophotometrically (2103 Envision Multilabel Reader, Perkin Elmer Waltham, MA, USA). The percentage survival was calculated as follows:

Survival (%) = (abs_sample_ − abs_pos.controle_)/(abs_neg.controle_ − abs_pos.controle_) × 100 (1)

Results were expressed as fold dilution at which 50% of the cells died (FD_50_).

### 2.4. Statistical Analysis

Supervised cluster analysis (Partial Least Squares Discriminant Analysis (PLS-DA)) was applied to evaluate the impact of lactose fermentation as a function of time on the metabolite patterns in the samples from NLD, LM-IT and LM-T subjects using Unscrambler software (CAMO A/S, Trondheim, Norway). In addition, associations between fermentation metabolites and cytotoxicity were studied. Differences in relative concentrations of volatile organic compounds and fecal water cytotoxicity between NLD, LM-IT and LM-T were evaluated using a Kruskall-Wallis-test followed by a Mann Whitney U-test. False discovery rate correction was applied to correct for multiple testing. Differences in absolute concentration of selected volatile organic compounds were evaluated using a general linear model with three between-subject factors (Time of incubation (0 h, 3 h, 6 h and 24 h), Group (NLD, LM-IT and LM-T) and Treatment (with and without lactose added)). The statistical level of significance was set to *p* < 0.05.

## 3. Results

### 3.1. Study Population

Between January 2013 and December 2014, 4742 ^13^C-lactose-breath tests have been performed in UZ Leuven. After screening, 92 subjects with a positive lactose breath test that lived in the proximity of Leuven were selected and were contacted. Sixty of them had reported symptoms whereas 32 subjects had no complaints during the test day. Finally, 11 LM-IT (6 m/5 f, age: 42 ± 12 years, BMI: 24.1 ± 2.1 kg/m^2^) and 8 LM-T (4 m/4 f, age: 40 ± 16 years, BMI: 23.5 ± 2.4 kg/m^2^) agreed to participate in the study and collected a fecal sample. In addition, 15 NLD (5 m/10 f, age: 30 ± 10 years, BMI: 23.4 ± 2.4 kg/m^2^) collected a fecal sample.

Cumulative ^13^CO_2_-excretion amounted to 20.5% ± 2.5% in NLD, while this was only 10.1% ± 3.3% in LM-IT and 12.7% ± 1.8% in LM-T. H_2_-excretion increased with 6.3 ± 7.8 ppm in NLD, 35.8 ± 47.4 ppm in LM-IT and 23.2 ± 26.8 ppm in LM-T. Two subjects in the NLD-group reported mild symptoms. No complaints were registered in the LM-T group whereas subjects in this LM-IT group reported at least one of the following symptoms: diarrhea (five subjects), flatulence (four subjects), cramps (two subjects), nausea (two subjects) and abdominal disturbances (two subjects).

### 3.2. Fecal Water Cytotoxicity in Samples from NLD, LM-IT and LM-T Subjects at Baseline

**Figure 1:**
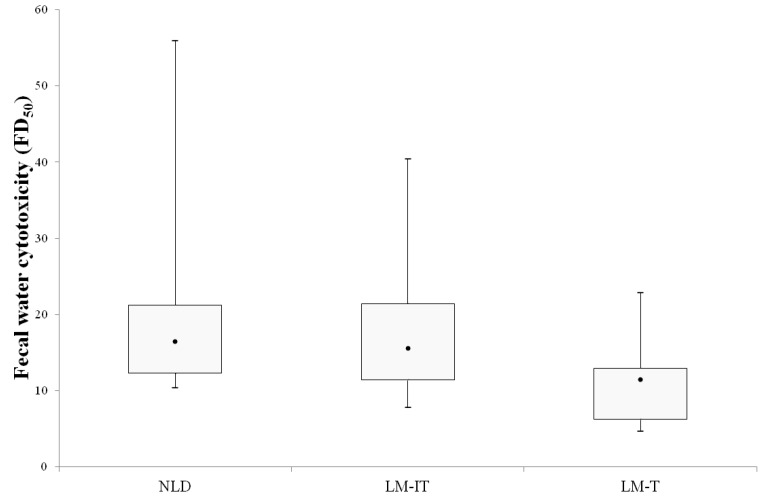
Fecal water toxicity in LM-IT, LM-T and NLD (median (Interquartile Range (IQR))) expressed as the fold dilution at which half of the cells died (FD_50_). NLD: normal lactose digestion; LM-IT: lactose malabsorption with abdominal complaints; LM-T: lactose malabsorption with without abdominal complaints.

The fecal samples displayed a large interindividual variation in cytotoxicity. Fecal water cytotoxicity was not different between the three groups (NLD: 16.4 (12.3–21.2); LM-IT: 15.5 (11.4–21.4); LM-T: 11.4 (6.3–12.9); Kruskall-Wallis *p* = 0.164) (Figure 1).

### 3.3. Metabolite Profiles in Fecal Samples from NLD, LM-IT and LM-T Subjects at Baseline

At baseline, the levels of the protein fermentation metabolites, dimethylsulfide (*p* = 0.009), isobutyrate (*p* = 0.028) and total BCFA (*p* = 0.041), were significantly higher in subjects with NLD compared to LM-IT (Table 1). Isobutyrate (*p* = 0.038), *p*-cresol (*p* = 0.012) and total BCFA (*p* = 0.037) were higher in NLD subjects compared to LM-T and *p*-cresol was higher in LM-IT compared to LM-T. Levels of SCFA, which result from carbohydrate fermentation, were not different between the three groups.

**Table 1:** Baseline concentrations of absolutely quantified metabolites in the LM-IT, LM-T and NLD (mean ± SD).

Metabolites	LM-IT	LM-T	NLD	*p*-Value
**Total SCFA (mM)**	213.2 (±118.4)	226.4 (±67.6)	188.0 (±68.8)	0.084
**Acetate (mM)**	162.8 (±98.4)	176.8 (±52.8)	141.6 (±53.6)	0.084
**Propionate (mM)**	33.2 (±16.4)	35.2 (±13.2)	34.8 (±15.2)	0.459
**Butyrate (mM)**	17.2 (±12.4)	14.4 (±8.4)	10.8 (±6.4)	0.072
**Total BCFA (mM)**	2.88 (±0.78) ^a^	2.71 (±1.71) ^a^	3.60 (±1.76) ^b^	0.037
**Isobutyrate (mM)**	1.95 (±0.60) ^a^	1.85 (±1.14) ^a^	2.49 (±1.11) ^b^	0.032
**Isovalerate (mM)**	0.92 (±0.46)	0.85 (±0.68)	1.16 (±0.72)	0.298
***p*-Cresol (µM)**	259 (±162) ^b^	173 (±127) ^a^	262 (±137) ^b^	0.035
**Indole (µM)**	19 (±12)	16 (±13)	18 (±14)	0.396
**Dimethylsulfide (µM)**	14 (±10) ^a^	15 (±7) ^ab^	16 (±8) ^b^	0.037

^a^, ^b^ values with different subscript within the same row were significantly different (*p* < 0.05). NLD: normal lactose digestion; LM-IT: lactose malabsorption with abdominal complaints; LM-T: lactose malabsorption with without abdominal complaints.

**Figure 2:**
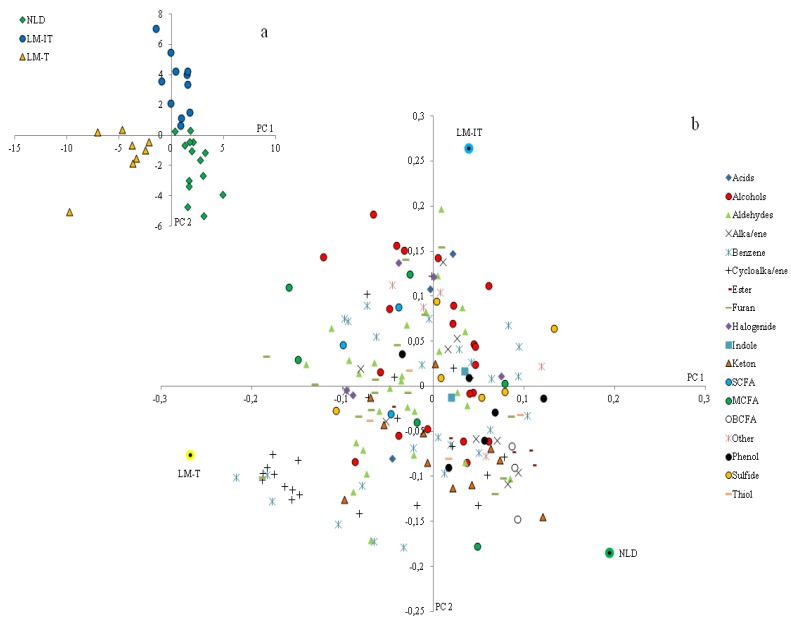
(**a**) Results of multivariate analysis of fecal metabolite profiles. Relative indices of the metabolites detected in the fecal samples collected by LM-IT, LM-T and NLD at baseline were used for a supervised clustering analysis by a partial least squares-discriminant analysis. The resulting loadings for principal components (PC) 1 and 2 are shown; (**b**) The bi-plot shows the metabolites classified according to chemical classes and the mean of the samples for each group of samples. NLD: normal lactose digestion; LM-IT: lactose malabsorption with abdominal complaints; LM-T: lactose malabsorption with without abdominal complaints.

PLS-DA of the baseline metabolite patterns measured in the samples derived from NLD, LM-IT and LM-T revealed separate clusters for each group (Figure 2a). SCFA, aldehydes and alcohols were more prevalent in the samples from subjects with LM-IT and LM-T. Ketones were associated with LM-IT and NLD whereas sulfides, alkanes and alkenes were associated with samples from subjects with LM-IT and NLD. Protein fermentation metabolites, such as indoles, branched chain fatty acids (BCFA) and phenols were more prevalent in NLD-samples, while cycloalkanes and cycloalkenes were more prevalent in LM-T-samples (Figure 2b). The relative concentration of 5-methyl-2-furancarboxaldehyde was significantly lower in NLD (0 (0–0)) compared to LM-IT (0.00096 (0–0.00194); *p* = 0.00146) and LM-T (0.00173 (0.00065–0.00328); *p* = 0.02267), whereas the relative concentration of an unidentified aldehyde was significantly higher in LM-IT (0.0039 (0–0.00677)) compared to NLD (0 (0–0); *p* = 0.00468)) and LM-T (0 (0–0); *p* < 0.001).

### 3.4. Metabolite Profiles after Incubation of Samples from NLD, LM-IT and LM-T with and without Lactose

**Figure 3:**
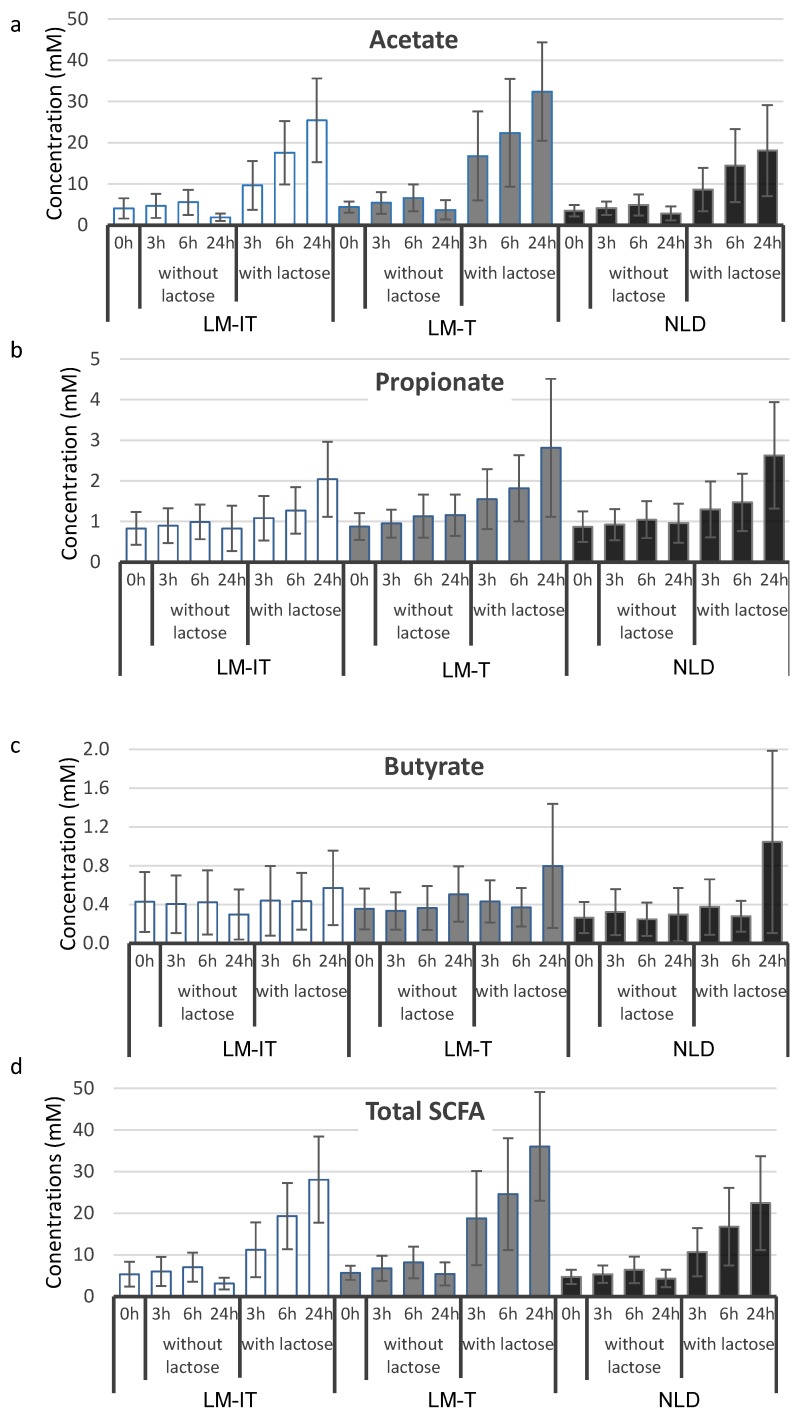
Mean concentrations of acetate (**a**), propionate (**b**), butyrate (**c**) and total SCFA (**d**) at baseline (0 h) and after incubation for 3 h, 6 h and 24 h with and without 6.25 mg of lactose in 1/40 diluted slurries from samples collected by subjects with LM-IT, LM-T or NLD. NLD: normal lactose digestion; LM-IT: lactose malabsorption with abdominal complaints; LM-T: lactose malabsorption with without abdominal complaints.

Concentrations of SCFA increased over time during incubation in each group (acetate: *p* < 0.001; propionate: *p* < 0.001; butyrate: *p* < 0.001) and were significantly higher after incubation with lactose compared to incubation without lactose (acetate: *p* < 0.001; propionate: *p* < 0.001; butyrate: *p* = 0.003) (Figure 3). After incubation with lactose, concentrations of acetate increased significantly more in the LM-IT and LM-T samples compared to the NLD samples (*p* < 0.001).

**Figure 4:**
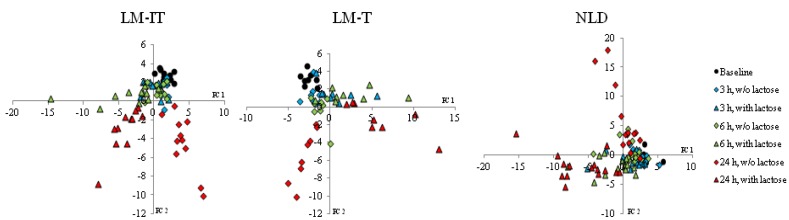
Results of multivariate analysis of fecal metabolite profiles. Relative indices of the metabolites detected in the fecal samples collected by LM-IT, LM-T and NLD at baseline and after incubation for 3 h, 6 h and 24 h with and without 6.25 mg of lactose were used for a supervised clustering analysis by a partial least squares-discriminant analysis. The resulting scores for principal components (PC) 1 and 2 are shown. Corresponding loading plots are presented in supplementary information. NLD: normal lactose digestion; LM-IT: lactose malabsorption with abdominal complaints; LM-T: lactose malabsorption with without abdominal complaints.

Incubation of fecal slurries with or without lactose clearly affected the metabolite patterns in the three groups (Figure 4). In each group incubation for 24 h without lactose was associated with a gradual increase in protein fermentation metabolites (sulfides, BCFA, phenols and indoles) and ketones, while incubation with lactose was associated with an increased production of SCFA.

**Figure 5:**
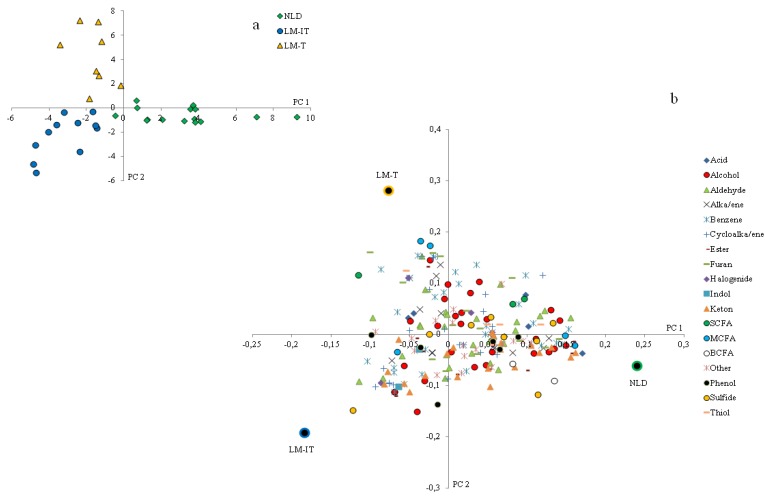
(**a**) Results of multivariate analysis of fecal metabolite profiles after 24 h incubation with lactose. Relative indices of the metabolites detected in the fecal samples collected by LM-IT, LM-T and NLD at baseline were used for a supervised clustering analysis by a partial least squares-discriminant analysis. The resulting loadings for principal components (PC) 1 and 2 are shown; (**b**) The bi-plot shows the metabolites divided in chemical classes and the mean of the samples for each intervention.

After 24 h incubation with lactose, the metabolite patterns in the three groups were clearly separated on cluster analysis (Figure 5). The production of the two volatile organic compounds that were significantly different between the three groups at baseline had increased after incubation with lactose but their relative concentrations remained significantly different. The relative concentration of 5-methyl-2-furancarboxaldehyde was significantly lower in NLD (0 (0–0)) compared to LM-IT (0.00202 (0.00052–0.00326); *p* = 0.00146) and LM-T (0.00272 (0.00235–0.00379); *p* = 0.02267), while the relative concentration of the unidentified aldehyde was significantly higher in LM-IT (0.0073 (0–0.01161)) compared to NLD (0 (0–0); *p* = 0.00042)) and LM-T (0 (0–0); *p* < 0.001).

**Figure 6:**
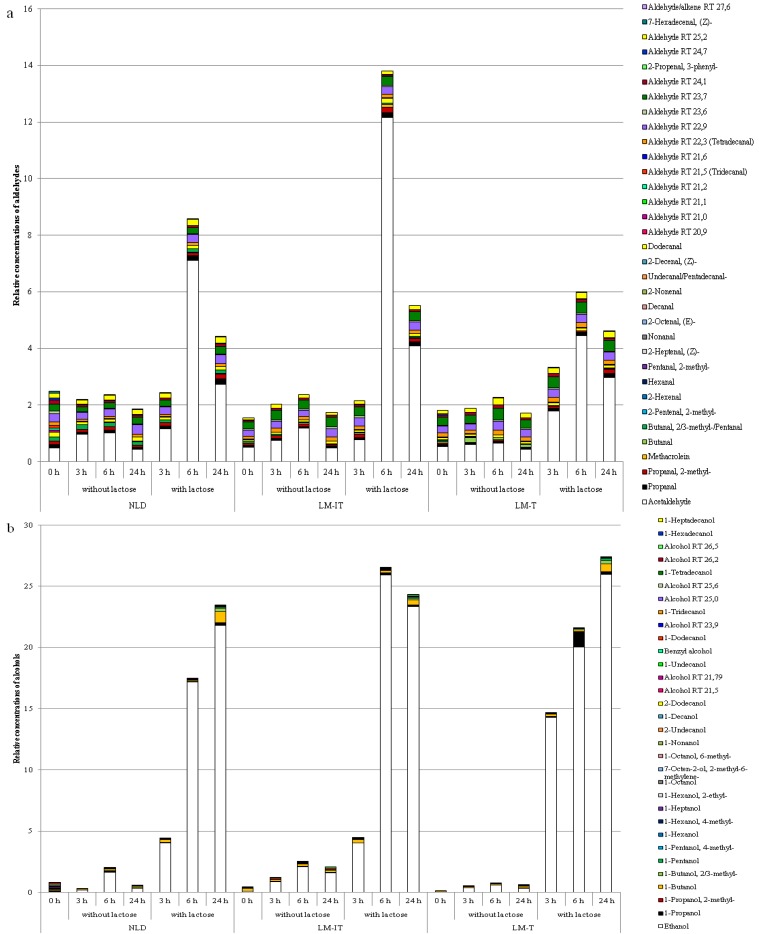
Relative concentrations of aldehydes (**a**) and alcohols (**b**) present in the NLD-, LM-IT- and LM-T-samples at baseline (0 h) or after incubation with or without lactose for 3 h, 6 h or 24 h. NLD: normal lactose digestion; LM-IT: lactose malabsorption with abdominal complaints; LM-T: lactose malabsorption with without abdominal complaints.

In addition, production of aldehydes and alcohols increased during fermentation of lactose (Figure 6). Most prevalent compounds in these chemical classes were acetaldehyde and ethanol, respectively. Remarkably, kinetics of aldehyde and alcohol production were higher in the samples from the LM-IT subjects. Levels of both groups of compounds were higher after 6 h of incubation in LM-IT samples compared to NLD and LM-T samples and leveled after 24 h. In the three groups of samples, levels of aldehydes were maximal after 6 h of incubation and decreased after 24 h.

### 3.5. Correlation between Fermentation Metabolites and Cytotoxicity

**Figure 7:**
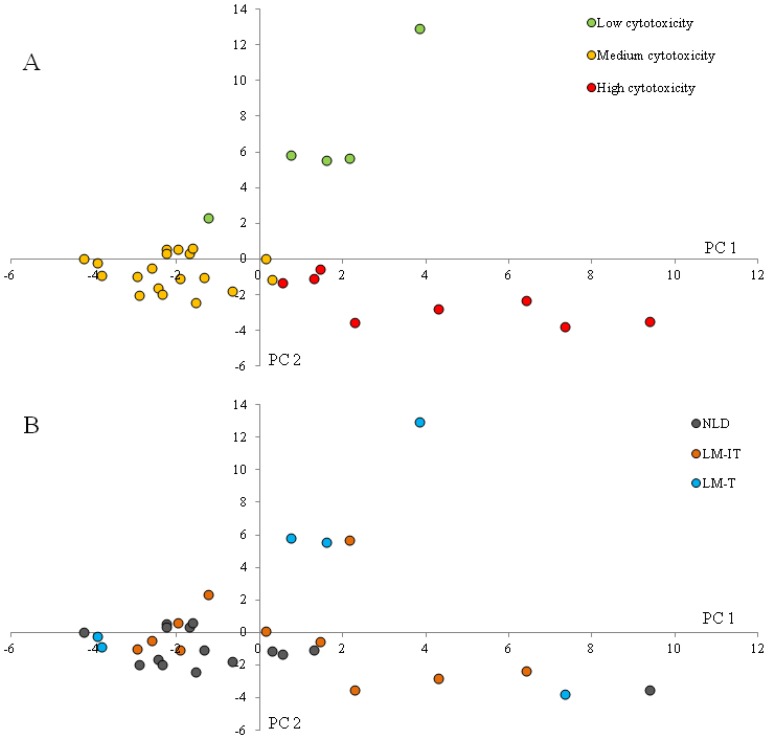

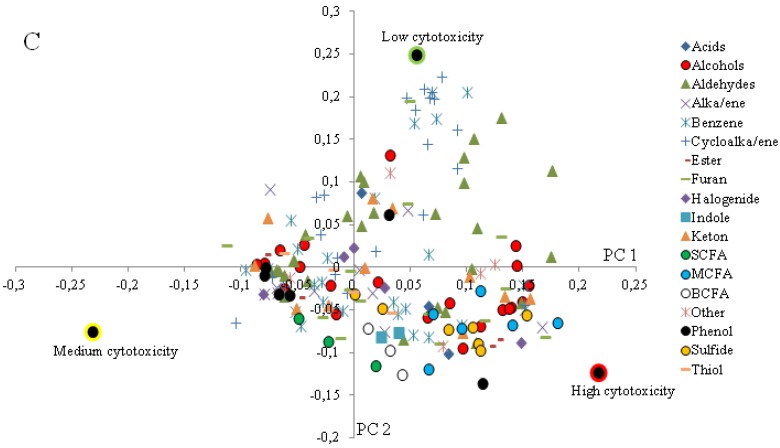
(**A**) Results of multivariate analysis of fecal metabolite profiles colored according to cytotoxicity and (**B**) colored according to lactose-digestibility status (LM-IT, LM-T and NLD). Relative indices of the metabolites detected in the fecal samples collected by LM-IT, LM-T and NLD at baseline were used for a supervised clustering analysis by a partial least squares-discriminant analysis according to cytotoxicity; (**C**) The resulting loadings for principal components (PC) 1 and 2 are shown. LM-IT: lactose malabsorption with abdominal complaints; LM-T: lactose malabsorption with without abdominal complaints.

To evaluate whether specific metabolites were associated with samples with high or low cytotoxicity, the samples were clustered according to cytotoxicity. Figure 7a shows clustering of the samples according to their level of cytotoxicity (low (FD_50_ < 10), medium (10 < FD_50_ < 20) or high (FD_50_ > 20)). In Figure 7b, the samples were recolored according to the subject group (NLD, LM-IT or LM-T). No clustering at all was observed which confirms that the cytotoxicity of the samples is not associated with the lactose digestibility status of the donor. Samples with high cytotoxicity were associated with protein fermentation metabolites such as sulfides, BCFA and indoles as well as with medium chain fatty acids. SCFA were associated with samples with medium cytotoxicity and cycloalkanes and cycloalkenes with samples with low cytotoxicity (Figure 7c). Aldehydes were more prevalent in samples with low and medium cytotoxicity, while alcohols were more abundant in samples with medium to high cytotoxicity.

## 4. Discussion

Not all subjects with lactose malabsorption experience undesirable effects such as bloating, cramps or abdominal pain and are to be considered as lactose-intolerant. Therefore lactose malabsorption and lactose intolerance are not synonymous and should not be interchanged. A systematic review that included the results of 18 studies found that 33%–97% of the subjects with confirmed lactose malabsorption in a hydrogen breath test, reported symptoms [4]. Potential factors that may contribute to the generation of symptoms include the orocecal transit time and hence the contact time of intestinal lactase with its substrate [5], visceral hypersensitivity [16], psychological factors [17] and the composition and activity of the microbiota [6,18]. This study evaluated the hypothesis that the lactose that remains unabsorbed in the small intestine is fermented in a different way in intolerant subjects compared to tolerant subjects and that the differences in fermentation metabolites might explain the occurrence of complaints. We compared the metabolite profiles of fecal samples obtained from subjects with and without lactose intolerance at baseline and after *in vitro* incubation with lactose. Fecal samples from subjects with normal lactose digestion were added as a control.

Analysis of the baseline samples revealed clear differences between the three groups of samples. Concentrations of protein fermentation metabolites were higher in subjects with normal lactose digestion compared to those with low lactose digestion. A lower degree of protein fermentation can be due to either a lower intake of protein [15,19] or to a higher supply of undigested carbohydrates to the colon increasing the ratio of carbohydrate-to-protein fermentation [20,21]. We can assume that particularly subjects with lactose intolerance avoid ingestion of dairy products such as milk, yoghurt and cheese. This might result in a lower protein intake and hence, lower protein fermentation, as dairy products are important sources of dietary protein. Information on macronutrient intake in lactose digesters and non-digesters is scarce. Carrocia *et al.* observed a significantly lower milk intake in intolerant lactose maldigesters and a non-significantly lower intake of cheese compared to lactose digesters and tolerant non-digesters. However, the relative protein intake was similar in the three groups [22].

Subjects that are tolerant to lactose malabsorption probably keep including dairy products in their diet. As the lactose is at least not completely digested, it enters the colon where it acts as a prebiotic and increases the level of carbohydrate fermentation. An increase in carbohydrate fermentation is a well-known mechanism of prebiotic administration to reduce the degree of protein fermentation. Indeed, the intestinal microbiota preferentially ferment carbohydrates as this process yields more energy than protein fermentation. Unfortunately, dietary intake was not assessed in this study, preventing us from confirming this hypothesis.

After incubation with lactose, the production of SCFA was higher in fecal samples from subjects with lactose malabsorption (both tolerant and intolerant) compared to subjects with a normal lactose digestion, confirming previous results [7]. Although several mechanisms that relate SCFA to abdominal discomfort have been proposed, it is rather unlikely that SCFA would accumulate in the colon and be responsible for the observed complaints in lactose intolerant subjects in view of the high capacity of the large intestine to absorb SCFA [7]. The use of a metabolomic approach enabled us to identify differences in the levels of several other metabolites including alcohols, aldehydes, ketones and protein fermentation metabolites in fecal samples.

In particular, levels of alcohols and aldehydes increased clearly more in the samples with lactose than in the samples without lactose. Whereas alcohol levels generally increased as a function of time, levels of aldehydes were highest after 6 h and decreased after 24 h suggesting that aldehydes were not end products of fermentation and were further converted. Most obvious reactions are a reduction to alcohols [23] or an oxidation to acids.

The levels of 5-methyl-2-furancarboxaldehyde (or 5-methyl furfural; CAS 620-02-0) were significantly higher in the samples from both groups with lactose-malabsorption compared to lactose digesters. This compound occurs in pepper and other plant sources and has previously been identified in fecal samples [24]. In addition, it is doubtless a secondary metabolite from carbohydrate metabolism [25]. Indeed, its concentration increased after incubation of the fecal samples with lactose. *In vitro* studies showed that methyl furfural is mutagenic and induces strand breaks in DNA [26]. In the loading plot corresponding to the clustering of the samples according to cytotoxicity, 5-methyl-2-furancarboxaldehyde is associated with the samples with highest cytotoxicity which may suggest that this compound contributes to the toxicity of the samples. However, as the levels were not different in samples from tolerant *versus* non-tolerant subjects, this compound is likely not involved in the generation of symptoms in lactose-intolerant subjects. Similarly, although the levels of the unidentified aldehyde were higher in the samples from lactose maldigesters, we assume that this compound is not involved in toxicity as it is more prevalent in the samples with low to medium cytotoxicity.

Our results suggest that fecal water cytotoxicity is not the major trigger and does not explain the occurrence of abdominal complaints in intolerant lactose malabsorbers as the fecal water cytotoxicity was not different between the three groups. Campbell *et al.* introduced the bacterial metabolic toxin hypothesis, stating that bacterial metabolites resulting from carbohydrate fermentation, such as alcohols, aldehydes, acids and ketones, play a role in the pathogenesis of lactose-intolerance as these metabolites might inhibit bacterial growth and affect eukaryotic cells [6]. A recent study in rats indicated that methylglyoxal, a metabolite produced by intestinal bacteria, could induce diarrhea, visceral hypersensitivity, headache as well as depression-like behaviors and might be involved in triggering symptoms of IBS. In the present study [27], we found that besides protein fermentation metabolites, in particular a number of alcohols were associated with higher cytotoxicity. In previous studies that associated fecal metabolite patterns to fecal water cytotoxicity, alcohols and medium chain fatty acids also contributed to increased toxicity [15,28] confirming Campbell’s hypothesis. However, we did not find evidence for toxicity of ketones as those metabolites were more prevalent in samples with low cytotoxicity.

It is important to realize that the toxicity of fecal water was measured on the baseline fecal samples. Assessment of cytotoxicity after incubation with lactose might have been more informative but was not possible due to analytical constraints. Fecal slurries had to be used in a 1/40 dilution in order not to overload the GC column. These suspensions were too diluted for cytotoxicity experiments as the median fold dilution that induced 50% of cell death ranged from 11.4 for the samples from LM-T subjects to 16.4 for the samples from NLD subjects. Alternatively, lactose-malabsorbing subjects could have been asked to consume a lactose-rich diet during a few days prior to the sample collection. However, the amount of lactose reaching the colon may vary between subjects and hampers the interpretation of the changes in fecal metabolite profiles.

In addition, the fermentation pattern of lactose was evaluated using fecal microbiota which does not necessarily reflect the microbiota in the proximal part of the colon where lactose is fermented *in vivo*. Cecal microbiota contains 100 less anaerobes than the fecal microbiota whereas facultative anaerobes constitute 25% of the microbiota in the cecum and only 1% in the feces [29]. As a consequence, the differences in fermentation patterns observed between the three groups might be slightly different from the differences in the proximal colon.

In the present study, we did not analyze the microbiota composition of the fecal samples as we hypothesized that differences in microbiota composition might explain differences in metabolite production but would not directly explain complaints. In addition, previous studies have already indicated that the microbiota composition did not differ between tolerant and intolerant subjects with lactose malabsorption [7] nor between subjects with normal lactose digestion and subjects with lactose malabsorption [30]. Nevertheless, it cannot be excluded that the bacterial techniques used in these studies (fluorescent *in situ* hybridization (FISH) and bacterial counting after plating, respectively) were not sensitive enough to detect subtle differences in microbiota composition and that current state-of-the-art techniques like next generation sequencing might be more informative.

## 5. Conclusions

At baseline, colonic fermentation differs in subjects with normal lactose digestion compared to subjects with lactose malabsorption with or without lactose intolerance. In addition, the functional capacity of the microbiota to process lactose is different between subjects with normal lactose digestion, lactose tolerant malabsorbers and lactose intolerant malabsorbers. However, these differences did not result in differences in fecal water cytotoxicity between the three groups. Consequently, it seems unlikely that induction of fecal water cytotoxicity is a major trigger for the occurrence of abdominal symptoms related to lactose malabsorption. Whether specific metabolites are able to trigger lactose intolerance through other mechanisms than induction of cytotoxicity remains to be investigated.

## Supplementary Files

Supplementary File 1
