# Supplementary material for: Contribution of Colonic Fermentation and Fecal Water Toxicity to the Pathophysiology of Lactose-Intolerance
Source: Nutrients. 2015 Sep 8;7(9):7505–22. doi: 10.3390/nu7095349 (PMC4586544; doi:10.3390/nu7095349)
Supplement: Supplementary File 1 [file nutrients-07-05349-s001.docx]

Supplementary Materials


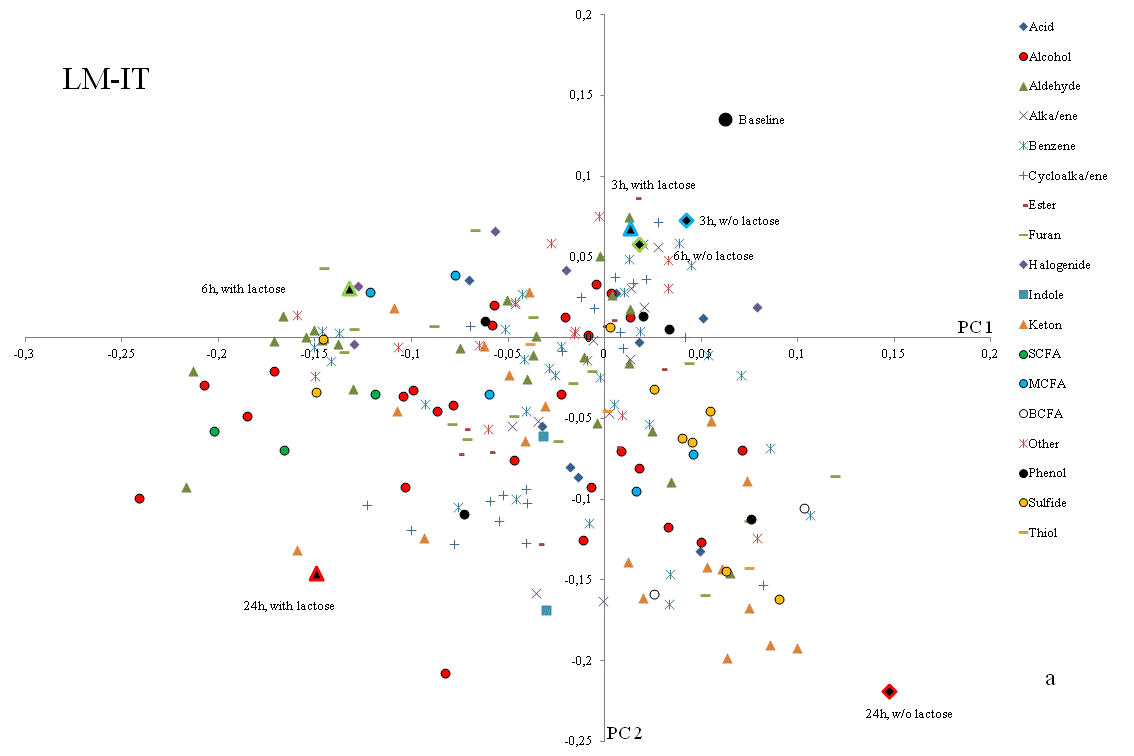


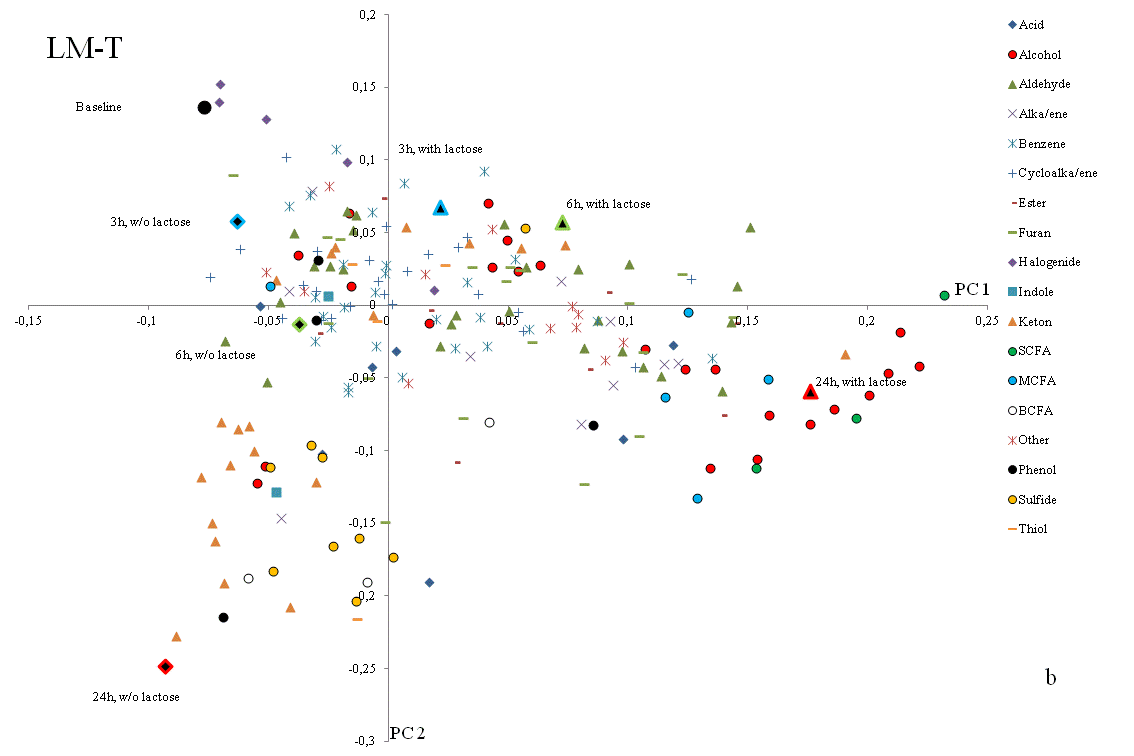


**Figure S1.** *Cont.*


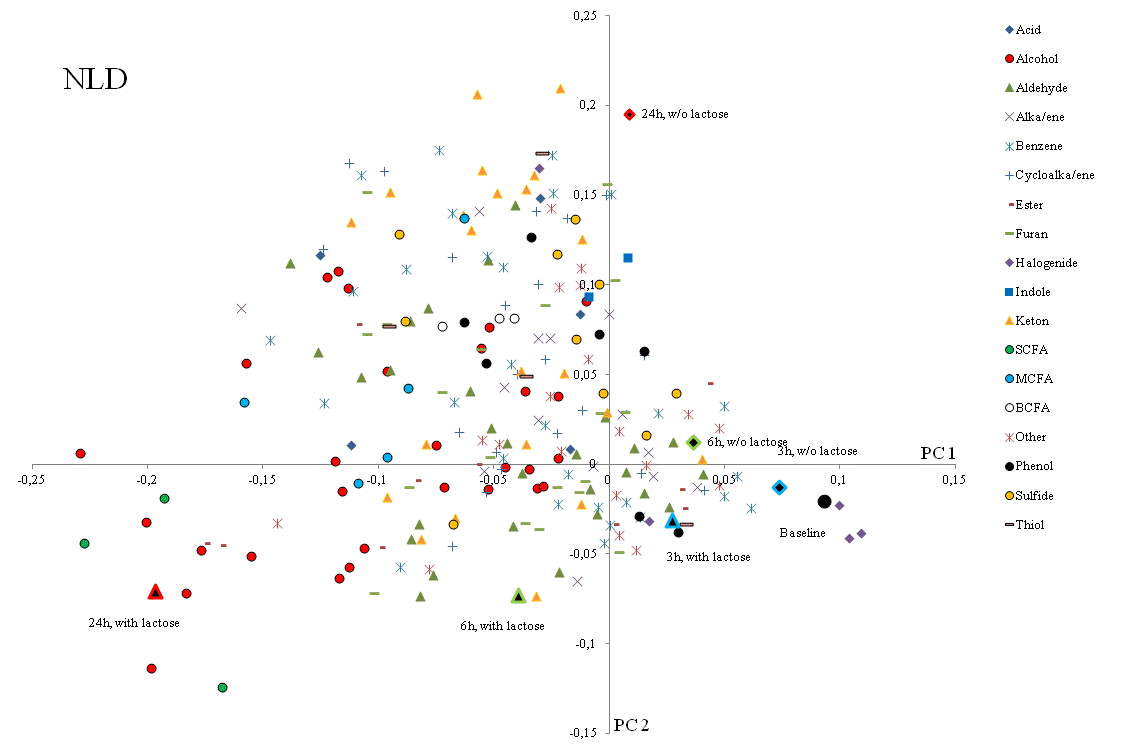


**Figure S1.** Results of multivariate analysis of fecal metabolite profiles. Relative indices of the metabolites detected in the fecal samples collected by LM-IT (**a**); LM-T (**b**) and
NLD (**c**) at baseline and after incubation for 3 h, 6 h and 24 h with and without 6.25 mg of lactose were used for a supervised clustering analysis by a partial least squares-discriminant analysis. The resulting loadings for principle components (PC) 1 and 2 are shown.

**Table S1.** Percentage occurrence and mean relative indices (I) of VOC in the fecal slurries at baseline and after incubation for 24h with and without lactose.

| **VOC** | **NLD** | | | | | | **LM-IT** | | | | | | **LM-T** | | | | | |
| --- | --- | --- | --- | --- | --- | --- | --- | --- | --- | --- | --- | --- | --- | --- | --- | --- | --- | --- |
|  |  |  | **without Lactose** | | **with Lactose** | |  |  | **without Lactose** | | **with Lactose** | |  |  | **without Lactose** | | **with Lactose** | |
|  | **0h** | | **24h** | | **24h** | | **0h** | | **24h** | | **24h** | | **0h** | | **24h** | | **24h** | |
|  | **I (×100)** | **%** | **I (×100)** | **%** | **I (×100)** | **%** | **I (×100)** | **%** | **I (×100)** | **%** | **I (×100)** | **%** | **I (×100)** | **%** | **I (×100)** | **%** | **I (×100)** | **%** |
| Pentane | 0.227 | 0 | 0.000 | 0 | 0.000 | 0 | 0.572 | 9 | 0.000 | 0 | 1.417 | 9 | 0.000 | 0 | 0.000 | 0 | 0.000 | 0 |
| Alka/ene RT 1,905 | 0.384 | 27 | 0.000 | 0 | 0.523 | 20 | 0.000 | 0 | 0.000 | 0 | 0.000 | 0 | 0.000 | 0 | 0.000 | 0 | 0.000 | 0 |
| Hydrogen sulfide | 0.000 | 7 | 0.715 | 13 | 1.420 | 27 | 0.000 | 0 | 1.924 | 27 | 0.000 | 0 | 0.000 | 0 | 0.775 | 25 | 0.000 | 0 |
| Oxetane, 3-(1-methylethyl)- | 0.351 | 0 | 0.000 | 0 | 0.000 | 0 | 0.495 | 18 | 0.000 | 0 | 0.210 | 9 | 0.000 | 0 | 0.000 | 0 | 0.000 | 0 |
| 1,3-Butadiene, 2-methyl- | 0.009 | 47 | 3.034 | 73 | 4.008 | 80 | 0.402 | 45 | 5.700 | 73 | 4.419 | 73 | 1.127 | 50 | 2.671 | 62.5 | 4.726 | 87.5 |
| 1-Butene, 2,3-dimethyl- | 16.628 | 7 | 0.042 | 7 | 0.044 | 7 | 0.000 | 0 | 0.245 | 18 | 0.000 | 0 | 0.034 | 12.5 | 0.530 | 12.5 | 1.121 | 12.5 |
| Ethyl ether | 6.522 | 73 | 123.818 | 80 | 58.707 | 73 | 5.123 | 64 | 42.933 | 64 | 58.443 | 82 | 120.853 | 100 | 183.433 | 87.5 | 23.925 | 87.5 |
| Methanethiol | 0.000 | 47 | 82.516 | 40 | 16.402 | 33 | 1.572 | 36 | 39.826 | 55 | 10.956 | 36 | 0.324 | 12.5 | 46.943 | 50 | 11.438 | 37.5 |
| Heptane | 0.019 | 0 | 0.000 | 0 | 0.000 | 0 | 0.000 | 0 | 0.000 | 0 | 0.743 | 9 | 0.000 | 0 | 0.000 | 0 | 0.000 | 0 |
| 3-Hexen-2-one | 0.000 | 7 | 0.000 | 0 | 0.000 | 0 | 0.000 | 0 | 0.000 | 0 | 0.000 | 0 | 0.000 | 0 | 0.000 | 0 | 0.000 | 0 |
| Cyclohexane | 0.000 | 0 | 0.000 | 0 | 0.000 | 0 | 0.000 | 0 | 0.000 | 0 | 0.000 | 0 | 0.030 | 12.5 | 0.000 | 0 | 0.000 | 0 |
| Benzonitrile | 48.586 | 0 | 0.000 | 0 | 0.000 | 0 | 0.000 | 0 | 0.000 | 0 | 0.000 | 0 | 0.000 | 0 | 0.000 | 0 | 0.000 | 0 |
| Acetaldehyde | 0.918 | 100 | 42.613 | 93 | 273.341 | 100 | 52.115 | 100 | 49.226 | 91 | 407.903 | 100 | 53.377 | 100 | 42.920 | 87.5 | 298.215 | 100 |
| Carbon disulfide | 14.663 | 53 | 1.655 | 40 | 2.048 | 67 | 0.412 | 18 | 6.097 | 45 | 1.595 | 55 | 2.585 | 50 | 3.641 | 50 | 1.326 | 50 |
| Dimethyl sulfide | 0.000 | 93 | 151.087 | 100 | 20.531 | 93 | 9.012 | 91 | 74.550 | 100 | 11.210 | 100 | 8.660 | 75 | 52.304 | 100 | 21.649 | 100 |
| Ethanethiol | 0.056 | 0 | 0.000 | 0 | 0.000 | 0 | 0.000 | 0 | 0.000 | 0 | 0.000 | 0 | 0.000 | 0 | 0.000 | 0 | 0.000 | 0 |
| 4-Methyl-2-hexene | 0.000 | 7 | 0.000 | 0 | 0.000 | 0 | 0.279 | 27 | 0.034 | 18 | 0.050 | 9 | 0.000 | 0 | 0.058 | 12.5 | 0.306 | 12.5 |
| 3-Hexen-2-one | 4.688 | 0 | 0.000 | 0 | 0.004 | 7 | 0.000 | 0 | 0.000 | 0 | 0.000 | 0 | 0.048 | 12.5 | 0.000 | 0 | 0.000 | 0 |
| Furan | 0.000 | 67 | 2.631 | 87 | 1.961 | 73 | 2.007 | 100 | 3.119 | 91 | 2.419 | 82 | 1.395 | 87.5 | 1.694 | 87.5 | 2.870 | 100 |
| Hexane, 2,4-dimethyl- | 12.500 | 0 | 0.000 | 0 | 0.000 | 0 | 0.009 | 9 | 0.375 | 9 | 0.317 | 9 | 0.000 | 0 | 0.000 | 0 | 0.000 | 0 |
| Propanal | 10.810 | 80 | 7.580 | 73 | 19.913 | 93 | 5.507 | 91 | 5.501 | 73 | 16.110 | 100 | 6.028 | 100 | 4.749 | 87.5 | 14.994 | 100 |
| Propanal, 2-methyl- | 0.000 | 73 | 7.368 | 73 | 15.163 | 100 | 8.221 | 91 | 6.741 | 64 | 10.660 | 82 | 5.636 | 87.5 | 3.406 | 62.5 | 13.673 | 100 |
| Acetic acid, methyl ester | 0.000 | 0 | 0.000 | 0 | 0.565 | 13 | 0.000 | 0 | 0.000 | 0 | 3.882 | 9 | 0.000 | 0 | 0.000 | 0 | 0.000 | 0 |
| 2-Propenal | 5.077 | 0 | 0.000 | 0 | 0.000 | 0 | 0.487 | 9 | 0.000 | 0 | 0.000 | 0 | 0.000 | 0 | 0.000 | 0 | 0.000 | 0 |
| Furan, 3-methyl- | 40.663 | 80 | 3.087 | 87 | 2.266 | 80 | 1.649 | 82 | 4.932 | 100 | 1.997 | 91 | 1.638 | 87.5 | 3.424 | 100 | 3.774 | 100 |
| Acetone | 4.467 | 87 | 23.216 | 60 | 95.414 | 100 | 39.072 | 91 | 42.224 | 82 | 98.081 | 100 | 37.985 | 87.5 | 38.088 | 87.5 | 109.027 | 100 |
| Furan, 2-methyl- | 0.000 | 60 | 1.164 | 67 | 0.481 | 60 | 0.094 | 45 | 0.168 | 45 | 0.428 | 45 | 0.104 | 50 | 0.119 | 50 | 0.158 | 50 |
| 2-Propenal, 2-methyl- | 0.000 | 0 | 0.000 | 0 | 0.129 | 7 | 0.000 | 0 | 0.000 | 0 | 0.000 | 0 | 0.000 | 0 | 0.000 | 0 | 0.000 | 0 |
| Methacrolein | 0.390 | 0 | 0.634 | 7 | 0.883 | 27 | 0.000 | 0 | 0.000 | 0 | 0.092 | 18 | 1.548 | 12.5 | 0.000 | 0 | 0.420 | 12.5 |
| Butanal | 1.034 | 7 | 0.000 | 0 | 2.334 | 53 | 0.625 | 9 | 0.320 | 9 | 1.877 | 55 | 2.674 | 12.5 | 10.617 | 37.5 | 0.849 | 25 |
| Furan, tetrahydro- | 0.000 | 47 | 2.076 | 53 | 8.062 | 80 | 5.014 | 73 | 8.261 | 73 | 12.775 | 91 | 1.849 | 37.5 | 0.128 | 12.5 | 5.561 | 62.5 |
| 2-Propanol, 2-methyl- | 0.031 | 0 | 0.000 | 0 | 0.000 | 0 | 0.000 | 0 | 0.000 | 0 | 0.000 | 0 | 0.000 | 0 | 0.000 | 0 | 0.000 | 0 |
| alpha-Pinene | 0.000 | 7 | 0.000 | 0 | 0.000 | 0 | 0.000 | 0 | 0.000 | 0 | 0.000 | 0 | 0.000 | 0 | 0.000 | 0 | 0.000 | 0 |
| Ethyl Acetate | 8.820 | 0 | 0.000 | 0 | 17.397 | 20 | 0.000 | 0 | 0.000 | 0 | 68.103 | 27 | 0.000 | 0 | 0.000 | 0 | 7.769 | 12.5 |
| Methylene Chloride | 0.041 | 33 | 23.018 | 47 | 6.628 | 40 | 19.023 | 45 | 6.940 | 73 | 6.186 | 73 | 47.537 | 37.5 | 14.132 | 75 | 13.428 | 62.5 |
| Benzene | 14.928 | 40 | 0.039 | 27 | 0.000 | 0 | 0.067 | 45 | 0.094 | 45 | 0.036 | 18 | 0.088 | 50 | 0.081 | 25 | 0.065 | 25 |
| Butanal, 2/3-methyl-/Pentanal | 4.262 | 53 | 13.184 | 47 | 11.824 | 33 | 5.084 | 45 | 1.913 | 18 | 6.591 | 27 | 6.457 | 50 | 0.000 | 0 | 3.045 | 25 |
| Furan, 2,5-dimethyl- | 2.408 | 60 | 0.354 | 60 | 0.261 | 27 | 0.176 | 73 | 0.235 | 73 | 0.056 | 18 | 0.106 | 50 | 0.011 | 12.5 | 0.061 | 25 |
| Ethanol | 0.034 | 20 | 31.199 | 53 | 2178.359 | 100 | 9.727 | 45 | 157.408 | 55 | 2332.272 | 100 | 0.000 | 0 | 33.463 | 75 | 2598.894 | 100 |
| 1,3-Cyclopentadiene, 5-(1,1-dimethylethyl)- | 1.590 | 20 | 0.142 | 20 | 0.000 | 0 | 0.000 | 0 | 0.011 | 18 | 0.000 | 0 | 0.035 | 62.5 | 0.010 | 12.5 | 0.000 | 0 |
| 2-Butanone | 0.067 | 47 | 4.968 | 53 | 14.660 | 20 | 1.705 | 45 | 15.051 | 55 | 23.866 | 27 | 9.961 | 62.5 | 7.971 | 12.5 | 10.925 | 62.5 |
| Furan, 2,4-dimethyl- | 0.000 | 7 | 0.000 | 0 | 0.000 | 0 | 0.000 | 0 | 0.000 | 0 | 0.000 | 0 | 0.115 | 12.5 | 0.000 | 0 | 0.000 | 0 |
| Propane, 2-methyl-1-nitro- | 0.000 | 0 | 0.201 | 7 | 0.000 | 0 | 0.000 | 0 | 0.000 | 0 | 0.000 | 0 | 0.000 | 0 | 0.000 | 0 | 0.000 | 0 |
| 2,3-Butanedione | 0.020 | 0 | 0.000 | 0 | 0.245 | 7 | 0.000 | 0 | 0.000 | 0 | 53.467 | 27 | 0.000 | 0 | 0.000 | 0 | 26.710 | 75 |
| 3,3-Dimethyl-6-methylenecyclohexene | 0.000 | 7 | 0.061 | 7 | 0.000 | 0 | 0.000 | 0 | 0.000 | 0 | 0.000 | 0 | 0.000 | 0 | 0.000 | 0 | 0.000 | 0 |
| Acetic acid ethenyl ester | 0.009 | 0 | 0.000 | 0 | 0.000 | 0 | 0.000 | 0 | 0.000 | 0 | 0.000 | 0 | 0.000 | 0 | 0.000 | 0 | 0.000 | 0 |
| alpha-Pinene | 2.249 | 7 | 0.280 | 27 | 0.067 | 7 | 0.040 | 9 | 0.601 | 27 | 4.230 | 27 | 1.483 | 12.5 | 1.013 | 50 | 1.317 | 25 |
| Acetonitrile | 10.213 | 33 | 0.676 | 13 | 2.794 | 53 | 24.747 | 82 | 17.996 | 45 | 21.264 | 55 | 17.395 | 62.5 | 19.010 | 75 | 15.009 | 62.5 |
| Trichloromethane | 4.873 | 87 | 6.915 | 100 | 4.097 | 67 | 7.276 | 91 | 8.181 | 91 | 5.972 | 82 | 402.160 | 87.5 | 153.971 | 100 | 190.262 | 87.5 |
| Toluene | 0.310 | 73 | 0.532 | 60 | 0.263 | 47 | 0.257 | 64 | 0.481 | 55 | 0.141 | 36 | 0.231 | 62.5 | 0.193 | 75 | 0.137 | 25 |
| 2-Pentenal, 2-methyl- | 0.004 | 7 | 0.224 | 7 | 0.000 | 0 | 0.675 | 18 | 0.474 | 27 | 0.000 | 0 | 0.078 | 12.5 | 0.000 | 0 | 0.000 | 0 |
| 2-Pentanone | 0.000 | 7 | 0.144 | 7 | 0.000 | 0 | 0.000 | 0 | 0.253 | 18 | 0.000 | 0 | 0.000 | 0 | 0.000 | 0 | 0.000 | 0 |
| 2-Butenal | 0.000 | 0 | 0.000 | 0 | 0.000 | 0 | 0.000 | 0 | 0.000 | 0 | 0.000 | 0 | 0.000 | 0 | 0.000 | 0 | 0.000 | 0 |
| 2-Butanone, 3-methyl- | 0.000 | 0 | 0.000 | 0 | 0.000 | 0 | 0.000 | 0 | 0.000 | 0 | 0.000 | 0 | 0.013 | 12.5 | 0.000 | 0 | 0.000 | 0 |
| 2-Acetyl-5-methylfuran | 0.047 | 0 | 0.000 | 0 | 0.000 | 0 | 0.000 | 0 | 0.000 | 0 | 0.000 | 0 | 0.005 | 12.5 | 0.000 | 0 | 0.000 | 0 |
| Camphene | 0.000 | 27 | 0.157 | 33 | 0.026 | 7 | 0.235 | 45 | 0.777 | 64 | 1.166 | 18 | 1.200 | 62.5 | 1.573 | 50 | 0.089 | 25 |
| Mercaptoacetone | 0.000 | 0 | 0.000 | 0 | 0.000 | 0 | 0.000 | 0 | 0.162 | 9 | 0.000 | 0 | 0.000 | 0 | 0.000 | 0 | 0.000 | 0 |
| 4-Hexen-3-one | 9.710 | 0 | 0.000 | 0 | 0.000 | 0 | 0.000 | 0 | 0.000 | 0 | 0.121 | 9 | 0.000 | 0 | 0.000 | 0 | 0.000 | 0 |
| Disulfide, dimethyl | 4.804 | 100 | 62.584 | 100 | 9.076 | 100 | 7.254 | 100 | 95.853 | 100 | 13.505 | 100 | 1.941 | 100 | 36.527 | 100 | 10.523 | 100 |
| 1-Propanol | 0.000 | 27 | 1.439 | 20 | 24.707 | 87 | 0.609 | 27 | 0.583 | 27 | 15.729 | 64 | 1.448 | 12.5 | 3.716 | 75 | 20.890 | 100 |
| Ethanone, 1-(2-methyl-1-cyclopenten-1-yl)- | 0.037 | 0 | 0.000 | 0 | 0.000 | 0 | 0.000 | 0 | 0.000 | 0 | 0.007 | 9 | 0.000 | 0 | 0.000 | 0 | 0.000 | 0 |
| 2-Hexenal | 0.000 | 7 | 0.040 | 7 | 0.000 | 0 | 0.000 | 0 | 0.000 | 0 | 0.000 | 0 | 0.480 | 12.5 | 0.613 | 12.5 | 0.000 | 0 |
| 2,3-Butanedione | 5.200 | 0 | 0.186 | 7 | 7.165 | 33 | 0.000 | 0 | 0.000 | 0 | 0.000 | 0 | 0.000 | 0 | 0.000 | 0 | 0.000 | 0 |
| 1-Propanol, 2-methyl- | 4.931 | 40 | 0.740 | 53 | 1.931 | 47 | 0.321 | 55 | 3.988 | 36 | 1.459 | 27 | 0.235 | 37.5 | 0.855 | 62.5 | 0.260 | 37.5 |
| Thiophene, 3-methyl- | 0.174 | 47 | 0.131 | 33 | 0.171 | 40 | 0.068 | 55 | 0.173 | 45 | 0.060 | 27 | 0.126 | 75 | 0.050 | 25 | 0.085 | 50 |
| beta-Pinene | 0.000 | 13 | 0.000 | 0 | 0.000 | 0 | 0.024 | 9 | 0.053 | 9 | 0.303 | 18 | 0.021 | 12.5 | 0.032 | 12.5 | 0.014 | 12.5 |
| 2-Pentanone, 3-methyl- | 0.000 | 0 | 0.361 | 13 | 0.765 | 7 | 0.000 | 0 | 0.497 | 9 | 0.000 | 0 | 0.000 | 0 | 0.000 | 0 | 0.000 | 0 |
| 2H-Pyran, 2-ethenyltetrahydro-2,6,6-trimethyl- | 0.000 | 0 | 0.000 | 0 | 0.000 | 0 | 0.000 | 0 | 0.000 | 0 | 0.000 | 0 | 0.000 | 0 | 0.112 | 12.5 | 0.000 | 0 |
| (+)-4-carene | 0.000 | 0 | 0.000 | 0 | 0.000 | 0 | 0.000 | 0 | 0.000 | 0 | 0.000 | 0 | 0.098 | 37.5 | 0.027 | 12.5 | 0.146 | 12.5 |
| Thiophene, 2-methyl- | 0.000 | 0 | 0.021 | 13 | 0.008 | 7 | 0.000 | 0 | 0.000 | 0 | 0.000 | 0 | 0.000 | 0 | 0.000 | 0 | 0.000 | 0 |
| Ethylbenzene | 0.117 | 0 | 0.000 | 0 | 0.069 | 13 | 0.000 | 0 | 0.000 | 0 | 0.024 | 9 | 0.000 | 0 | 0.039 | 12.5 | 0.018 | 12.5 |
| Hexanal | 0.000 | 13 | 0.202 | 7 | 0.357 | 7 | 0.034 | 18 | 0.000 | 0 | 0.000 | 0 | 0.469 | 25 | 0.042 | 12.5 | 0.000 | 0 |
| 2-n-Butyl furan | 5.062 | 0 | 0.000 | 0 | 0.000 | 0 | 0.002 | 9 | 0.000 | 0 | 0.000 | 0 | 0.000 | 0 | 0.000 | 0 | 0.000 | 0 |
| 3-Carene | 5.001 | 47 | 0.316 | 27 | 0.753 | 13 | 0.828 | 55 | 1.413 | 55 | 0.694 | 36 | 3.566 | 75 | 3.318 | 50 | 3.112 | 75 |
| o/p-Xylene | 0.002 | 47 | 0.020 | 27 | 0.009 | 7 | 0.004 | 27 | 0.051 | 36 | 0.020 | 18 | 0.027 | 37.5 | 0.003 | 12.5 | 0.011 | 12.5 |
| Furan, 2-ethyl-5-methyl- | 0.000 | 7 | 0.012 | 13 | 0.000 | 0 | 0.000 | 0 | 0.000 | 0 | 0.000 | 0 | 0.000 | 0 | 0.000 | 0 | 0.000 | 0 |
| 2,3-Butanedione/2-Pentanone | 0.000 | 0 | 0.008 | 7 | 0.000 | 0 | 0.000 | 0 | 0.000 | 0 | 1.005 | 36 | 0.000 | 0 | 0.000 | 0 | 0.089 | 12.5 |
| Methyl ethyl disulfide | 0.008 | 0 | 0.014 | 7 | 0.000 | 0 | 0.042 | 27 | 0.079 | 27 | 0.027 | 36 | 0.012 | 12.5 | 0.023 | 12.5 | 0.000 | 0 |
| 2-Hexanone, 3-methyl-/2-Pentanone, 3-methyl- | 0.000 | 7 | 2.523 | 60 | 0.000 | 0 | 0.000 | 0 | 1.517 | 45 | 0.045 | 18 | 0.060 | 12.5 | 3.444 | 75 | 0.000 | 0 |
| Butanoic acid, ethyl ester | 0.002 | 0 | 0.000 | 0 | 0.000 | 0 | 0.000 | 0 | 0.000 | 0 | 0.000 | 0 | 0.000 | 0 | 0.000 | 0 | 0.345 | 25 |
| .alpha.-Phellandrene | 0.000 | 7 | 0.000 | 0 | 0.012 | 13 | 0.000 | 0 | 0.000 | 0 | 0.000 | 0 | 0.014 | 12.5 | 0.000 | 0 | 0.010 | 12.5 |
| .beta.-Myrcene | 5.114 | 0 | 0.000 | 0 | 0.000 | 0 | 0.000 | 0 | 0.000 | 0 | 0.000 | 0 | 0.000 | 0 | 0.000 | 0 | 0.000 | 0 |
| Methane, bromodichloro- | 0.000 | 80 | 0.000 | 0 | 0.000 | 0 | 0.159 | 91 | 0.006 | 9 | 0.005 | 18 | 0.141 | 100 | 0.000 | 0 | 0.000 | 0 |
| p-Xylene | 0.044 | 0 | 0.000 | 0 | 0.000 | 0 | 0.000 | 0 | 0.001 | 9 | 0.004 | 9 | 0.002 | 12.5 | 0.000 | 0 | 0.000 | 0 |
| 1,3-Cyclohexadiene, 1-methyl-4-(1-methylethyl)- | 0.000 | 40 | 0.081 | 27 | 0.083 | 33 | 0.062 | 45 | 0.043 | 36 | 0.114 | 45 | 0.305 | 62.5 | 0.317 | 62.5 | 0.290 | 37.5 |
| 1,3,8-p-Menthatriene | 5.098 | 0 | 0.000 | 0 | 0.000 | 0 | 0.000 | 0 | 0.000 | 0 | 0.000 | 0 | 0.000 | 0 | 0.000 | 0 | 0.000 | 0 |
| Benzene, (1-methylethyl)- | 0.000 | 13 | 0.035 | 7 | 0.012 | 7 | 0.019 | 18 | 0.036 | 18 | 0.000 | 0 | 0.000 | 0 | 0.000 | 0 | 0.000 | 0 |
| 1,3,7-Octatriene, 2,7-dimethyl- | 0.449 | 0 | 0.000 | 0 | 0.000 | 0 | 0.000 | 0 | 0.000 | 0 | 0.000 | 0 | 0.000 | 0 | 0.000 | 0 | 0.000 | 0 |
| D-Limonene | 0.000 | 67 | 1.549 | 67 | 1.188 | 53 | 0.594 | 91 | 0.641 | 82 | 1.481 | 91 | 1.159 | 87.5 | 0.885 | 75 | 0.858 | 100 |
| 2-Hexanone, 4-methyl- | 0.000 | 0 | 0.000 | 0 | 0.000 | 0 | 0.000 | 0 | 0.010 | 9 | 0.000 | 0 | 0.000 | 0 | 0.000 | 0 | 0.000 | 0 |
| 2-Cyclopenten-1-one, 2,3,5-trimethyl-4-methylene- | 0.000 | 0 | 0.002 | 7 | 0.000 | 0 | 0.000 | 0 | 0.000 | 0 | 0.000 | 0 | 0.000 | 0 | 0.002 | 12.5 | 0.000 | 0 |
| Pentane | 0.051 | 0 | 0.081 | 7 | 0.000 | 0 | 0.000 | 0 | 0.000 | 0 | 0.000 | 0 | 0.000 | 0 | 0.000 | 0 | 0.000 | 0 |
| Cyclopropyl carbinol | 0.000 | 7 | 0.000 | 0 | 0.000 | 0 | 0.000 | 0 | 0.000 | 0 | 0.000 | 0 | 0.000 | 0 | 0.100 | 12.5 | 0.000 | 0 |
| 3,4-Dimethylthiophene | 0.000 | 0 | 0.000 | 0 | 0.000 | 0 | 0.000 | 0 | 0.000 | 0 | 0.000 | 0 | 0.003 | 12.5 | 0.000 | 0 | 0.009 | 25 |
| Pentanal, 2-methyl- | 0.000 | 0 | 0.011 | 7 | 0.000 | 0 | 0.000 | 0 | 0.000 | 0 | 0.000 | 0 | 0.000 | 0 | 0.000 | 0 | 0.000 | 0 |
| Butane, 2-methoxy-2-methyl- | 9.201 | 0 | 0.000 | 0 | 0.000 | 0 | 0.000 | 0 | 0.000 | 0 | 0.000 | 0 | 0.000 | 0 | 0.000 | 0 | 0.000 | 0 |
| 1-Butanol | 0.000 | 93 | 9.428 | 100 | 87.381 | 100 | 20.794 | 100 | 14.233 | 100 | 39.121 | 100 | 6.606 | 100 | 10.107 | 100 | 62.442 | 100 |
| Benzene, 1-ethyl-x-methyl- RT 9,4 | 0.000 | 0 | 0.000 | 0 | 0.000 | 0 | 0.000 | 0 | 0.000 | 0 | 0.000 | 0 | 0.007 | 12.5 | 0.000 | 0 | 0.000 | 0 |
| Cycloalka/ene RT 9,5 | 5.352 | 0 | 0.000 | 0 | 0.000 | 0 | 0.000 | 0 | 0.000 | 0 | 0.000 | 0 | 0.042 | 12.5 | 0.000 | 0 | 0.000 | 0 |
| Furan, 2-pentyl- | 0.012 | 47 | 0.027 | 40 | 0.046 | 53 | 0.022 | 36 | 0.038 | 55 | 0.020 | 27 | 0.054 | 75 | 0.026 | 37.5 | 0.022 | 25 |
| Disulfide, methyl propyl | 0.005 | 13 | 0.088 | 20 | 0.022 | 20 | 0.007 | 9 | 0.019 | 9 | 0.000 | 0 | 0.000 | 0 | 0.114 | 50 | 0.000 | 0 |
| Benzene, tert-butyl- | 5.577 | 13 | 0.000 | 0 | 0.000 | 0 | 0.012 | 27 | 0.011 | 18 | 0.000 | 0 | 0.000 | 0 | 0.000 | 0 | 0.000 | 0 |
| 1,4-Cyclohexadiene, 1-methyl-4-(1-methylethyl)- | 0.002 | 73 | 0.349 | 67 | 0.314 | 67 | 0.163 | 82 | 0.192 | 82 | 0.344 | 73 | 0.546 | 87.5 | 0.618 | 87.5 | 0.530 | 75 |
| 1-Pentanone, 1-(4-methylphenyl)- | 0.018 | 7 | 0.000 | 0 | 0.000 | 0 | 0.000 | 0 | 0.000 | 0 | 0.000 | 0 | 0.000 | 0 | 0.000 | 0 | 0.000 | 0 |
| Benzene, x,x,x-trimethyl- RT 9,9 | 0.000 | 27 | 0.208 | 67 | 0.076 | 40 | 0.024 | 45 | 0.212 | 55 | 0.154 | 82 | 0.048 | 37.5 | 0.099 | 62.5 | 0.077 | 50 |
| 3-Carene | 5.523 | 0 | 0.000 | 0 | 0.000 | 0 | 0.000 | 0 | 0.000 | 0 | 0.000 | 0 | 0.000 | 0 | 0.000 | 0 | 0.007 | 12.5 |
| Benzene, 1-methoxy-4-methyl- | 5.880 | 80 | 0.118 | 73 | 0.133 | 80 | 0.084 | 82 | 0.194 | 91 | 0.165 | 91 | 0.075 | 50 | 0.121 | 87.5 | 0.162 | 87.5 |
| Thiophene, 2,4-dimethyl- | 5.615 | 73 | 0.496 | 87 | 0.559 | 87 | 0.490 | 91 | 0.663 | 82 | 0.448 | 73 | 0.488 | 100 | 0.452 | 87.5 | 0.495 | 100 |
| 1-Butanol, 2/3-methyl- | 0.000 | 40 | 5.942 | 80 | 26.412 | 93 | 0.547 | 55 | 9.170 | 100 | 15.882 | 100 | 0.087 | 12.5 | 6.322 | 87.5 | 30.085 | 100 |
| Heptanal | 0.000 | 0 | 0.000 | 0 | 0.000 | 0 | 0.059 | 9 | 0.000 | 0 | 0.000 | 0 | 0.000 | 0 | 0.000 | 0 | 0.000 | 0 |
| Benzene, 1-methyl-x-(1-methylethyl)- RT 10,5 | 0.005 | 0 | 0.000 | 0 | 0.000 | 0 | 0.000 | 0 | 0.000 | 0 | 0.000 | 0 | 0.000 | 0 | 0.000 | 0 | 0.000 | 0 |
| Styrene | 7.963 | 13 | 0.006 | 13 | 1.344 | 13 | 0.003 | 9 | 0.000 | 0 | 0.003 | 9 | 0.004 | 12.5 | 0.009 | 12.5 | 0.006 | 12.5 |
| Benzene, 1-methyl-x-(1-methylethyl)- RT 10,6 | 0.000 | 100 | 4.087 | 93 | 3.343 | 100 | 2.178 | 100 | 2.989 | 82 | 2.575 | 91 | 5.570 | 100 | 5.821 | 75 | 7.032 | 87.5 |
| Benzene, 1-ethyl-2,4-dimethyl- | 0.128 | 0 | 0.000 | 0 | 0.000 | 0 | 0.000 | 0 | 0.000 | 0 | 0.000 | 0 | 0.000 | 0 | 0.061 | 12.5 | 0.000 | 0 |
| (+)-4-Carene RT 10,8 | 5.782 | 47 | 0.276 | 53 | 0.190 | 47 | 0.115 | 73 | 0.127 | 55 | 0.340 | 64 | 0.336 | 75 | 0.407 | 75 | 0.111 | 62.5 |
| Benzene, x,x,x-trimethyl- RT 11,0/Benzene,  1-ethyl-x-methyl- RT 11,1 | 0.000 | 80 | 0.182 | 53 | 0.103 | 53 | 0.291 | 91 | 0.198 | 73 | 0.293 | 100 | 0.494 | 100 | 0.304 | 87.5 | 0.174 | 25 |
| Cyclohexene, 1-methyl-4-(1-methylethylidene)- | 0.008 | 0 | 0.095 | 7 | 0.000 | 0 | 0.000 | 0 | 0.000 | 0 | 0.005 | 9 | 0.000 | 0 | 0.000 | 0 | 0.000 | 0 |
| Disulfide, methyl 2-propenyl | 0.000 | 7 | 0.032 | 13 | 0.027 | 13 | 0.000 | 0 | 0.000 | 0 | 0.000 | 0 | 0.000 | 0 | 0.103 | 37.5 | 0.012 | 12.5 |
| 2,4-Dithiapentane | 0.000 | 0 | 0.016 | 7 | 0.000 | 0 | 0.000 | 0 | 0.006 | 9 | 0.000 | 0 | 0.000 | 0 | 0.000 | 0 | 0.000 | 0 |
| Benzene, 1-ethyl-2-methyl- | 5.646 | 0 | 0.000 | 0 | 0.000 | 0 | 0.000 | 0 | 0.000 | 0 | 0.000 | 0 | 0.000 | 0 | 0.000 | 0 | 0.000 | 0 |
| Oxepine, 2,7-dimethyl- | 5.686 | 7 | 0.017 | 7 | 0.026 | 13 | 0.000 | 0 | 0.000 | 0 | 0.000 | 0 | 0.000 | 0 | 0.000 | 0 | 0.000 | 0 |
| 1-Pentanol | 0.000 | 27 | 0.285 | 27 | 12.701 | 100 | 0.334 | 27 | 1.017 | 45 | 7.282 | 91 | 0.030 | 37.5 | 0.603 | 50 | 16.668 | 87.5 |
| Thiocyanic acid, methyl ester | 5.718 | 0 | 0.046 | 7 | 0.000 | 0 | 0.000 | 0 | 0.744 | 9 | 0.000 | 0 | 0.000 | 0 | 0.000 | 0 | 0.000 | 0 |
| Bromochloronitromethane | 5.661 | 87 | 0.001 | 7 | 0.001 | 7 | 0.063 | 91 | 0.002 | 9 | 0.000 | 0 | 0.052 | 75 | 0.002 | 12.5 | 0.000 | 0 |
| Benzene, 1-methyl-3-(1-methylethyl)- | 0.002 | 53 | 0.036 | 20 | 0.068 | 40 | 0.023 | 18 | 0.006 | 9 | 0.000 | 0 | 0.101 | 25 | 0.188 | 37.5 | 0.129 | 25 |
| Cyclohexanone | 0.000 | 7 | 0.004 | 7 | 0.000 | 0 | 0.000 | 0 | 0.000 | 0 | 0.000 | 0 | 0.000 | 0 | 0.000 | 0 | 0.000 | 0 |
| Benzene, tert-butyl- | 0.000 | 0 | 0.033 | 7 | 0.006 | 7 | 0.000 | 0 | 0.000 | 0 | 0.000 | 0 | 0.000 | 0 | 0.000 | 0 | 0.000 | 0 |
| 2-Butanone, 3-hydroxy- | 0.022 | 0 | 0.000 | 0 | 0.119 | 13 | 0.000 | 0 | 0.000 | 0 | 0.347 | 18 | 0.000 | 0 | 0.000 | 0 | 0.041 | 25 |
| Cyclohexene, 3-methyl-6-(1-methylethylidene)- | 0.000 | 7 | 0.043 | 7 | 0.030 | 7 | 0.000 | 0 | 0.000 | 0 | 0.000 | 0 | 0.047 | 12.5 | 0.005 | 12.5 | 0.000 | 0 |
| 1-Penten-3-one, 2-methyl- | 0.000 | 0 | 0.000 | 0 | 0.000 | 0 | 0.000 | 0 | 0.000 | 0 | 0.000 | 0 | 0.000 | 0 | 0.000 | 0 | 0.000 | 0 |
| 1-Pentanol, 4-methyl- | 0.000 | 0 | 0.014 | 7 | 0.015 | 7 | 0.000 | 0 | 0.000 | 0 | 0.000 | 0 | 0.000 | 0 | 0.000 | 0 | 0.000 | 0 |
| 2-Heptenal, (Z)- | 5.787 | 0 | 0.000 | 0 | 0.000 | 0 | 0.000 | 0 | 0.000 | 0 | 0.000 | 0 | 0.000 | 0 | 0.000 | 0 | 0.000 | 0 |
| .alpha.-Methylstyrene | 0.108 | 20 | 0.007 | 13 | 0.013 | 20 | 0.044 | 27 | 0.048 | 9 | 0.038 | 18 | 0.061 | 25 | 0.044 | 12.5 | 0.042 | 12.5 |
| Benzene, 1-ethyl-x-methyl- RT 12,6/Benzene,  x,x,x-trimethyl- RT 12,6 | 0.000 | 67 | 0.239 | 67 | 0.156 | 67 | 0.086 | 73 | 0.121 | 64 | 0.137 | 91 | 0.165 | 100 | 0.157 | 75 | 0.148 | 75 |
| 5-Hepten-2-one, 6-methyl- | 0.003 | 0 | 0.000 | 0 | 0.000 | 0 | 0.000 | 0 | 0.000 | 0 | 0.010 | 9 | 0.015 | 12.5 | 0.000 | 0 | 0.000 | 0 |
| Allyl Isothiocyanate | 0.000 | 7 | 0.018 | 7 | 0.015 | 7 | 0.000 | 0 | 0.030 | 9 | 0.000 | 0 | 0.054 | 25 | 0.065 | 25 | 0.056 | 12.5 |
| Indane | 5.910 | 0 | 0.000 | 0 | 0.000 | 0 | 0.000 | 0 | 0.000 | 0 | 0.008 | 9 | 0.000 | 0 | 0.000 | 0 | 0.000 | 0 |
| 1-Hexanol | 0.000 | 27 | 0.210 | 40 | 2.333 | 93 | 0.614 | 73 | 1.046 | 73 | 2.283 | 100 | 0.178 | 37.5 | 0.506 | 50 | 4.175 | 87.5 |
| Hexane, 1,1'-oxybis- | 8.061 | 0 | 0.000 | 0 | 0.000 | 0 | 0.000 | 0 | 0.000 | 0 | 0.000 | 0 | 0.138 | 25 | 0.000 | 0 | 0.000 | 0 |
| Dimethyl trisulfide | 0.011 | 93 | 37.625 | 93 | 6.210 | 100 | 2.453 | 100 | 13.290 | 100 | 2.723 | 100 | 0.997 | 100 | 21.447 | 100 | 2.754 | 100 |
| Furan, 2-methyl-5-(methylthio)- | 0.000 | 20 | 0.082 | 47 | 0.088 | 40 | 0.000 | 0 | 0.104 | 55 | 0.033 | 45 | 0.000 | 0 | 0.072 | 37.5 | 0.031 | 50 |
| Keton RT 14,3 (2-Nonanone/2-Pentanone, 4-hydroxy-4-methyl-) | 0.011 | 0 | 0.062 | 20 | 0.306 | 20 | 0.000 | 0 | 0.155 | 36 | 0.017 | 9 | 0.000 | 0 | 0.019 | 12.5 | 0.009 | 12.5 |
| Nonanal | 0.005 | 7 | 0.045 | 7 | 0.019 | 13 | 0.000 | 0 | 0.000 | 0 | 0.013 | 9 | 0.032 | 25 | 0.066 | 25 | 0.000 | 0 |
| 3-Octanol/1-Hexanol, 4-methyl- | 0.000 | 7 | 0.008 | 7 | 0.000 | 0 | 0.000 | 0 | 0.000 | 0 | 0.007 | 9 | 0.009 | 12.5 | 0.000 | 0 | 0.000 | 0 |
| Propanoic acid, propyl ester | 0.000 | 0 | 0.000 | 0 | 0.000 | 0 | 0.000 | 0 | 0.000 | 0 | 0.000 | 0 | 0.000 | 0 | 0.006 | 12.5 | 0.000 | 0 |
| Cyclohexanecarboxylic acid, ethyl ester | 0.002 | 0 | 0.000 | 0 | 0.000 | 0 | 0.000 | 0 | 0.000 | 0 | 0.000 | 0 | 0.000 | 0 | 0.000 | 0 | 0.000 | 0 |
| Furan, tetrahydro-2,2,5,5-tetramethyl- | 0.000 | 7 | 0.013 | 13 | 0.036 | 20 | 0.006 | 9 | 0.019 | 18 | 0.001 | 9 | 0.012 | 12.5 | 0.008 | 12.5 | 0.027 | 25 |
| Benzene, 1,2,4,5-tetramethyl- | 0.012 | 0 | 0.000 | 0 | 0.000 | 0 | 0.000 | 0 | 0.000 | 0 | 0.000 | 0 | 0.000 | 0 | 0.000 | 0 | 0.000 | 0 |
| 2-Octenal, (E)- | 0.013 | 7 | 0.000 | 0 | 0.000 | 0 | 0.000 | 0 | 0.000 | 0 | 0.000 | 0 | 0.000 | 0 | 0.000 | 0 | 0.000 | 0 |
| o-Isopropenyltoluene | 6.403 | 7 | 0.237 | 13 | 0.196 | 20 | 0.008 | 9 | 0.019 | 9 | 0.192 | 27 | 0.487 | 37.5 | 0.327 | 25 | 0.467 | 37.5 |
| Benzene, 1,3-bis(1,1-dimethylethyl)- | 6.598 | 67 | 0.231 | 53 | 0.177 | 53 | 0.216 | 82 | 0.388 | 73 | 0.383 | 73 | 0.263 | 62.5 | 0.274 | 62.5 | 0.079 | 62.5 |
| Benzene, 1-methyl-4-(1-methylethenyl)- | 0.048 | 73 | 0.735 | 73 | 0.629 | 73 | 0.115 | 73 | 0.114 | 45 | 0.287 | 64 | 0.529 | 75 | 0.533 | 75 | 0.307 | 62.5 |
| Methane, tribromo-/Dibromonitromethane | 0.002 | 67 | 0.001 | 7 | 0.003 | 7 | 0.041 | 45 | 0.021 | 27 | 0.000 | 0 | 0.017 | 37.5 | 0.000 | 0 | 0.000 | 0 |
| cis-Linaloloxide/2-Furanmethanol,  5-ethenyltetrahydro-.alpha.,.alpha.,5-trimethyl-, trans- | 0.000 | 7 | 0.004 | 7 | 0.017 | 20 | 0.000 | 0 | 0.000 | 0 | 0.000 | 0 | 0.162 | 25 | 0.095 | 25 | 0.159 | 25 |
| 1-Butene, 4-isothiocyanato- | 6.316 | 0 | 0.000 | 0 | 0.000 | 0 | 0.000 | 0 | 0.000 | 0 | 0.000 | 0 | 0.000 | 0 | 0.000 | 0 | 0.000 | 0 |
| 1-Heptanol | 0.000 | 7 | 0.052 | 27 | 0.903 | 73 | 0.120 | 55 | 0.090 | 18 | 0.459 | 91 | 0.096 | 37.5 | 0.083 | 50 | 1.073 | 100 |
| Propanoic acid | 0.000 | 0 | 0.000 | 0 | 0.000 | 0 | 0.803 | 9 | 1.307 | 9 | 5.728 | 9 | 0.000 | 0 | 0.000 | 0 | 0.000 | 0 |
| Keton RT 15,8 (2-Decanone) | 33.018 | 0 | 0.000 | 0 | 0.040 | 7 | 0.000 | 0 | 0.000 | 0 | 0.000 | 0 | 0.000 | 0 | 0.000 | 0 | 0.000 | 0 |
| Acetic acid | 0.114 | 100 | 21.245 | 100 | 209.076 | 100 | 36.284 | 100 | 10.616 | 100 | 291.403 | 100 | 43.920 | 100 | 19.401 | 100 | 346.551 | 100 |
| Furfural | 0.000 | 47 | 0.109 | 33 | 0.164 | 53 | 0.081 | 55 | 0.038 | 18 | 0.142 | 82 | 0.172 | 75 | 0.151 | 62.5 | 0.226 | 62.5 |
| Ethanone, 1-(4-methylphenyl)- | 0.000 | 0 | 0.000 | 0 | 0.000 | 0 | 0.000 | 0 | 0.005 | 9 | 0.000 | 0 | 0.000 | 0 | 0.004 | 12.5 | 0.007 | 12.5 |
| Furan, 2-[(methylthio)methyl]- | 6.753 | 0 | 0.022 | 13 | 0.018 | 27 | 0.000 | 0 | 0.000 | 0 | 0.018 | 36 | 0.000 | 0 | 0.004 | 12.5 | 0.016 | 25 |
| 1-Hexanol, 2-ethyl- | 0.000 | 100 | 0.538 | 100 | 0.518 | 100 | 0.332 | 100 | 1.426 | 100 | 0.586 | 100 | 0.250 | 100 | 0.418 | 100 | 0.465 | 100 |
| Decanal | 0.000 | 0 | 0.000 | 0 | 0.000 | 0 | 0.014 | 9 | 0.019 | 9 | 0.000 | 0 | 0.038 | 12.5 | 0.029 | 12.5 | 0.000 | 0 |
| Copaene | 0.000 | 0 | 0.000 | 0 | 0.000 | 0 | 0.000 | 0 | 0.000 | 0 | 0.000 | 0 | 0.000 | 0 | 0.000 | 0 | 0.000 | 0 |
| Benzofuran | 0.000 | 0 | 0.000 | 0 | 0.000 | 0 | 0.000 | 0 | 0.000 | 0 | 0.002 | 9 | 0.000 | 0 | 0.000 | 0 | 0.000 | 0 |
| 2-Hepten-1-ol, (E)- | 0.000 | 0 | 0.000 | 0 | 0.000 | 0 | 0.000 | 0 | 0.000 | 0 | 0.000 | 0 | 0.000 | 0 | 0.000 | 0 | 0.000 | 0 |
| Alkaan RT 17,0 | 0.000 | 0 | 0.084 | 7 | 0.045 | 13 | 0.101 | 18 | 0.000 | 0 | 0.006 | 9 | 0.000 | 0 | 0.000 | 0 | 0.000 | 0 |
| 2-Nonanol | 0.000 | 0 | 0.000 | 0 | 0.000 | 0 | 0.000 | 0 | 0.000 | 0 | 0.000 | 0 | 0.000 | 0 | 0.000 | 0 | 0.000 | 0 |
| Furan, 2-ethyl-5-methyl- | 0.006 | 0 | 0.000 | 0 | 0.006 | 7 | 0.000 | 0 | 0.000 | 0 | 0.000 | 0 | 0.038 | 37.5 | 0.101 | 37.5 | 0.063 | 25 |
| Pyrrole | 0.326 | 7 | 0.012 | 13 | 0.000 | 0 | 0.000 | 0 | 0.000 | 0 | 0.000 | 0 | 0.000 | 0 | 0.000 | 0 | 0.000 | 0 |
| unknown RT 17.20 ion 126/193 | 0.000 | 93 | 0.250 | 87 | 0.371 | 87 | 0.236 | 82 | 0.225 | 82 | 0.295 | 73 | 0.287 | 100 | 0.184 | 87.5 | 0.469 | 87.5 |
| Ionone | 7.172 | 0 | 0.000 | 0 | 0.000 | 0 | 0.000 | 0 | 0.000 | 0 | 0.000 | 0 | 0.033 | 12.5 | 0.091 | 12.5 | 0.054 | 12.5 |
| Benzaldehyde | 0.020 | 87 | 0.276 | 80 | 1.144 | 93 | 0.332 | 100 | 0.284 | 73 | 1.474 | 100 | 0.207 | 75 | 0.208 | 62.5 | 0.524 | 100 |
| 2-Nonenal | 26.957 | 13 | 0.000 | 0 | 0.026 | 7 | 0.026 | 9 | 0.025 | 9 | 0.032 | 18 | 0.007 | 25 | 0.013 | 25 | 0.008 | 25 |
| Propanoic acid | 6.901 | 100 | 26.814 | 100 | 102.580 | 100 | 22.298 | 91 | 20.259 | 91 | 73.087 | 91 | 26.634 | 100 | 36.327 | 100 | 119.539 | 100 |
| 1-Octanol | 0.015 | 53 | 0.190 | 60 | 0.861 | 93 | 0.261 | 64 | 0.325 | 73 | 0.793 | 82 | 0.218 | 62.5 | 0.293 | 75 | 0.981 | 100 |
| Cyclohexanemethanol | 0.008 | 13 | 0.119 | 27 | 0.160 | 20 | 0.000 | 0 | 0.012 | 9 | 0.011 | 9 | 0.032 | 12.5 | 0.168 | 25 | 0.028 | 12.5 |
| unknown 155/163/193 | 0.000 | 7 | 0.030 | 7 | 0.033 | 7 | 0.105 | 27 | 0.076 | 36 | 0.173 | 36 | 0.024 | 25 | 0.072 | 25 | 0.031 | 25 |
| 3-Cyclohexen-1-ol, 1-methyl-4-(1-methylethyl)- | 45.315 | 0 | 0.000 | 0 | 0.000 | 0 | 0.000 | 0 | 0.000 | 0 | 0.000 | 0 | 0.107 | 25 | 0.045 | 12.5 | 0.000 | 0 |
| Propanoic acid, 2-methyl- | 0.000 | 100 | 60.435 | 100 | 54.119 | 100 | 33.538 | 100 | 47.667 | 100 | 38.052 | 100 | 31.238 | 100 | 67.283 | 100 | 37.119 | 100 |
| 2-Furancarboxaldehyde, 5-methyl- | 0.053 | 0 | 0.000 | 0 | 0.000 | 0 | 0.123 | 55 | 0.157 | 64 | 0.207 | 73 | 0.294 | 87.5 | 0.286 | 87.5 | 0.500 | 87.5 |
| Propanoic acid, 2,2-dimethyl- | 0.000 | 27 | 0.071 | 20 | 0.043 | 20 | 0.186 | 55 | 0.274 | 55 | 0.217 | 45 | 0.066 | 37.5 | 0.387 | 25 | 0.347 | 37.5 |
| Bicyclo[3.1.1]hept-2-ene, 2,6-dimethyl-6-(4-methyl-3-pentenyl)- | 0.000 | 0 | 0.000 | 0 | 0.000 | 0 | 0.000 | 0 | 0.000 | 0 | 0.000 | 0 | 0.010 | 12.5 | 0.000 | 0 | 0.000 | 0 |
| Caryophyllene | 0.044 | 0 | 0.000 | 0 | 0.000 | 0 | 0.000 | 0 | 0.000 | 0 | 0.399 | 18 | 0.000 | 0 | 0.000 | 0 | 0.000 | 0 |
| Alkaan RT 18,44 (Hexadecane) | 0.069 | 13 | 0.118 | 13 | 0.131 | 13 | 0.158 | 9 | 0.000 | 0 | 0.029 | 9 | 0.037 | 12.5 | 0.000 | 0 | 0.041 | 12.5 |
| 3-Cyclohexen-1-ol, 4-methyl-1-(1-methylethyl)-, (R)- | 0.038 | 20 | 0.200 | 13 | 0.170 | 27 | 0.017 | 18 | 0.000 | 0 | 0.114 | 36 | 0.048 | 12.5 | 0.089 | 12.5 | 0.065 | 12.5 |
| 2-Undecanone | 0.035 | 13 | 1.003 | 60 | 0.173 | 27 | 0.008 | 9 | 1.026 | 64 | 0.078 | 18 | 0.000 | 0 | 0.627 | 100 | 0.137 | 12.5 |
| Ethanone, 1-(2-methyl-1-cyclopenten-1-yl)- | 0.046 | 7 | 0.063 | 7 | 0.000 | 0 | 0.000 | 0 | 0.000 | 0 | 0.000 | 0 | 0.000 | 0 | 0.000 | 0 | 0.004 | 12.5 |
| Undecanal/Pentadecanal- | 0.000 | 7 | 0.031 | 7 | 0.021 | 7 | 0.000 | 0 | 0.000 | 0 | 0.000 | 0 | 0.000 | 0 | 0.000 | 0 | 0.000 | 0 |
| Benzofuran, 2-methyl- | 0.000 | 0 | 0.006 | 7 | 0.015 | 7 | 0.000 | 0 | 0.000 | 0 | 0.000 | 0 | 0.000 | 0 | 0.000 | 0 | 0.014 | 25 |
| 7-Octen-2-ol, 2-methyl-6-methylene- | 0.055 | 0 | 0.000 | 0 | 0.006 | 7 | 0.000 | 0 | 0.000 | 0 | 0.000 | 0 | 0.039 | 12.5 | 0.026 | 12.5 | 0.052 | 12.5 |
| 1-Octanol, 6-methyl- | 80.452 | 33 | 0.152 | 40 | 0.106 | 53 | 0.066 | 45 | 0.041 | 36 | 0.090 | 55 | 0.054 | 62.5 | 0.010 | 25 | 0.029 | 12.5 |
| Butanoic acid | 0.522 | 100 | 93.332 | 100 | 304.871 | 100 | 124.794 | 100 | 89.130 | 100 | 226.380 | 100 | 102.498 | 100 | 143.979 | 100 | 319.978 | 100 |
| Cyclohexanol, 5-methyl-2-(1-methylethyl)-,  (1.alpha.,2.beta.,5.alpha.)-(.+/-.)- | 0.116 | 60 | 0.695 | 60 | 0.782 | 67 | 0.564 | 45 | 0.569 | 73 | 1.003 | 55 | 0.109 | 25 | 0.145 | 12.5 | 0.765 | 62.5 |
| 2-Decenal, (Z)- | 0.000 | 7 | 0.555 | 13 | 0.239 | 7 | 0.063 | 9 | 0.000 | 0 | 0.066 | 9 | 0.173 | 12.5 | 0.017 | 12.5 | 0.105 | 25 |
| 5,7-Octadien-2-ol, 2,6-dimethyl- | 0.216 | 0 | 0.021 | 13 | 0.039 | 13 | 0.000 | 0 | 0.000 | 0 | 0.000 | 0 | 0.000 | 0 | 0.063 | 12.5 | 0.159 | 12.5 |
| Benzeneacetaldehyde | 7.244 | 40 | 0.198 | 27 | 0.269 | 33 | 0.114 | 45 | 0.057 | 18 | 0.053 | 18 | 0.058 | 37.5 | 0.041 | 25 | 0.038 | 12.5 |
| 1-Nonanol | 7.262 | 80 | 0.297 | 80 | 0.999 | 93 | 0.180 | 100 | 0.274 | 73 | 0.522 | 100 | 0.172 | 87.5 | 0.351 | 100 | 0.720 | 100 |
| Acetophenone | 0.000 | 73 | 0.238 | 60 | 0.338 | 67 | 0.102 | 73 | 0.200 | 73 | 0.182 | 73 | 0.126 | 75 | 0.141 | 87.5 | 0.207 | 87.5 |
| Bicyclo[2.2.1]heptane, 7,7-dimethyl-2-methylene- | 0.001 | 0 | 0.000 | 0 | 0.000 | 0 | 0.000 | 0 | 0.000 | 0 | 0.000 | 0 | 0.057 | 12.5 | 0.055 | 12.5 | 0.085 | 12.5 |
| 2/3-Furanmethanol | 0.000 | 7 | 0.000 | 0 | 0.000 | 0 | 0.000 | 0 | 0.000 | 0 | 0.000 | 0 | 0.000 | 0 | 0.000 | 0 | 0.000 | 0 |
| Disulfide, methyl (methylthio)methyl | 94.811 | 0 | 0.030 | 7 | 0.000 | 0 | 0.000 | 0 | 0.000 | 0 | 0.000 | 0 | 0.000 | 0 | 0.000 | 0 | 0.000 | 0 |
| Butanoic acid, 3-methyl- | 87.283 | 93 | 119.766 | 87 | 96.367 | 100 | 37.852 | 82 | 110.023 | 82 | 78.362 | 91 | 50.917 | 87.5 | 93.412 | 75 | 71.992 | 87.5 |
| Butanoic acid, 2-methyl- | 0.000 | 93 | 109.739 | 87 | 104.546 | 93 | 72.607 | 91 | 116.347 | 100 | 69.457 | 82 | 68.630 | 100 | 145.450 | 87.5 | 63.682 | 87.5 |
| Benzaldehyde, 4-hydroxy- | 0.584 | 0 | 0.000 | 0 | 0.004 | 7 | 0.000 | 0 | 0.000 | 0 | 0.000 | 0 | 0.000 | 0 | 0.000 | 0 | 0.000 | 0 |
| 3-Cyclohexene-1-methanol, .alpha.,.alpha.4-trimethyl- | 0.404 | 73 | 0.638 | 53 | 0.809 | 93 | 0.370 | 64 | 0.163 | 45 | 0.167 | 73 | 0.740 | 75 | 0.796 | 75 | 1.019 | 100 |
| Lang alkaan RT 19,9 (Nonadecane) | 0.019 | 20 | 1.548 | 13 | 1.085 | 27 | 0.106 | 18 | 0.011 | 9 | 0.118 | 18 | 0.003 | 12.5 | 0.053 | 12.5 | 0.000 | 0 |
| Furan, 2-ethyl-5-methyl- | 0.000 | 27 | 0.002 | 7 | 0.009 | 13 | 0.017 | 27 | 0.000 | 0 | 0.005 | 9 | 0.000 | 0 | 0.000 | 0 | 0.000 | 0 |
| Butanoic acid, 2,2-dimethyl- | 0.000 | 0 | 0.000 | 0 | 0.000 | 0 | 0.189 | 9 | 0.190 | 9 | 0.143 | 9 | 0.000 | 0 | 0.000 | 0 | 0.000 | 0 |
| Ethanone, 1-(2-furanyl)- | 0.000 | 0 | 0.000 | 0 | 0.009 | 7 | 0.000 | 0 | 0.000 | 0 | 0.012 | 9 | 0.000 | 0 | 0.000 | 0 | 0.000 | 0 |
| 2-Thiophenecarboxaldehyde | 0.000 | 0 | 0.000 | 0 | 0.000 | 0 | 0.000 | 0 | 0.005 | 9 | 0.000 | 0 | 0.000 | 0 | 0.000 | 0 | 0.000 | 0 |
| 2-Undecanol | 15.804 | 0 | 0.169 | 7 | 0.269 | 13 | 0.000 | 0 | 0.319 | 9 | 0.059 | 9 | 0.000 | 0 | 0.000 | 0 | 0.000 | 0 |
| Dodecanal | 0.000 | 100 | 13.890 | 93 | 11.381 | 100 | 5.697 | 100 | 8.110 | 100 | 8.060 | 91 | 8.283 | 100 | 9.002 | 100 | 10.663 | 100 |
| Caryophyllene | 0.711 | 0 | 0.000 | 0 | 0.000 | 0 | 0.000 | 0 | 0.000 | 0 | 0.068 | 9 | 0.000 | 0 | 0.000 | 0 | 0.000 | 0 |
| unknown RT 20.65 ion 138/193 | 0.000 | 87 | 0.838 | 87 | 0.696 | 93 | 0.787 | 82 | 0.591 | 82 | 0.587 | 82 | 0.274 | 100 | 0.549 | 100 | 0.535 | 87.5 |
| 1,3,6,10-Dodecatetraene, 3,7,11-trimethyl-, (Z,E)- | 0.000 | 0 | 0.000 | 0 | 0.000 | 0 | 0.012 | 9 | 0.009 | 9 | 0.035 | 18 | 0.000 | 0 | 0.000 | 0 | 0.034 | 12.5 |
| Aldehyde/alkene RT 20,4 | 0.038 | 0 | 0.000 | 0 | 0.000 | 0 | 0.000 | 0 | 0.000 | 0 | 0.000 | 0 | 0.000 | 0 | 0.000 | 0 | 0.000 | 0 |
| 2-Dodecanone | 47.479 | 13 | 0.198 | 33 | 0.059 | 20 | 0.005 | 9 | 0.234 | 27 | 0.013 | 9 | 0.019 | 12.5 | 0.068 | 25 | 0.023 | 12.5 |
| Pentanoic acid | 0.033 | 100 | 52.240 | 100 | 149.573 | 100 | 42.020 | 100 | 27.415 | 100 | 56.462 | 100 | 47.898 | 100 | 63.212 | 100 | 72.237 | 100 |
| 3,5-Dimethylanisole | 0.000 | 7 | 0.086 | 7 | 0.025 | 7 | 0.000 | 0 | 0.000 | 0 | 0.000 | 0 | 0.000 | 0 | 0.000 | 0 | 0.000 | 0 |
| Benzofuran, 4,7-dimethyl- | 0.000 | 0 | 0.000 | 0 | 0.002 | 7 | 0.000 | 0 | 0.000 | 0 | 0.000 | 0 | 0.000 | 0 | 0.000 | 0 | 0.000 | 0 |
| .alpha.-Farnesene | 0.000 | 0 | 0.000 | 0 | 0.000 | 0 | 0.129 | 18 | 0.176 | 18 | 0.105 | 9 | 0.000 | 0 | 0.000 | 0 | 0.293 | 25 |
| Benzene, 2-(1,3-butadienyl)-1,3,5-trimethyl- | 0.019 | 0 | 0.000 | 0 | 0.000 | 0 | 0.000 | 0 | 0.000 | 0 | 0.010 | 9 | 0.009 | 12.5 | 0.000 | 0 | 0.000 | 0 |
| 1-Decanol | 0.081 | 7 | 0.014 | 7 | 0.000 | 0 | 0.000 | 0 | 0.098 | 18 | 0.089 | 9 | 0.000 | 0 | 0.000 | 0 | 0.000 | 0 |
| Aldehyde RT 20,9 | 0.000 | 13 | 0.068 | 13 | 0.103 | 7 | 0.000 | 0 | 0.000 | 0 | 0.000 | 0 | 0.000 | 0 | 0.000 | 0 | 0.000 | 0 |
| Benzene, 1-(1,5-dimethyl-4-hexenyl)-4-methyl- | 7.660 | 0 | 0.000 | 0 | 0.000 | 0 | 0.000 | 0 | 0.000 | 0 | 0.010 | 9 | 0.016 | 12.5 | 0.028 | 25 | 0.070 | 25 |
| Aldehyde RT 21,0 | 0.007 | 60 | 0.424 | 53 | 0.444 | 67 | 0.135 | 45 | 0.123 | 27 | 0.192 | 45 | 0.240 | 62.5 | 0.137 | 50 | 0.392 | 87.5 |
| Naphthalene, 1,2-dihydro-1,1,6-trimethyl- | 0.000 | 7 | 0.009 | 7 | 0.014 | 7 | 0.000 | 0 | 0.000 | 0 | 0.004 | 9 | 0.013 | 12.5 | 0.056 | 25 | 0.034 | 12.5 |
| 3-Heptanone | 0.000 | 0 | 0.000 | 0 | 0.000 | 0 | 0.000 | 0 | 0.000 | 0 | 0.000 | 0 | 0.003 | 12.5 | 0.000 | 0 | 0.000 | 0 |
| Aldehyde RT 21,1 | 0.000 | 0 | 0.000 | 0 | 0.000 | 0 | 0.013 | 9 | 0.000 | 0 | 0.000 | 0 | 0.076 | 25 | 0.088 | 25 | 0.050 | 12.5 |
| 2-Dodecanol | 0.008 | 0 | 0.037 | 7 | 0.000 | 0 | 0.000 | 0 | 0.026 | 9 | 0.000 | 0 | 0.000 | 0 | 0.035 | 12.5 | 0.000 | 0 |
| Methyl Salicylate/*Benzaldehyde, 4-methyl-* | 0.003 | 13 | 0.000 | 0 | 0.000 | 0 | 0.012 | 18 | 0.000 | 0 | 0.000 | 0 | 0.027 | 25 | 0.000 | 0 | 0.000 | 0 |
| Ethanone, 1-(4-methylphenyl)- | 7.518 | 7 | 0.017 | 13 | 0.024 | 27 | 0.003 | 9 | 0.000 | 0 | 0.000 | 0 | 0.000 | 0 | 0.015 | 25 | 0.028 | 25 |
| Aldehyde RT 21,2 | 0.000 | 20 | 0.053 | 13 | 0.078 | 13 | 0.074 | 18 | 0.108 | 18 | 0.079 | 27 | 0.000 | 0 | 0.000 | 0 | 0.183 | 37.5 |
| Benzeneacetic acid, ethyl ester | 0.019 | 0 | 0.000 | 0 | 0.099 | 47 | 0.000 | 0 | 0.000 | 0 | 0.045 | 36 | 0.000 | 0 | 0.000 | 0 | 0.030 | 37.5 |
| Ethanone, 1-(2,4-dimethylphenyl)- | 0.500 | 7 | 0.019 | 13 | 0.043 | 7 | 0.000 | 0 | 0.004 | 9 | 0.000 | 0 | 0.004 | 12.5 | 0.000 | 0 | 0.014 | 12.5 |
| Dodecane, 2,6,11-trimethyl-/Eiscosane/Hexadecane | 8.330 | 20 | 0.591 | 27 | 0.672 | 33 | 0.041 | 9 | 0.079 | 18 | 0.219 | 27 | 0.076 | 25 | 0.087 | 25 | 0.208 | 12.5 |
| Pentanoic acid, 4-methyl- | 0.015 | 87 | 6.842 | 93 | 8.282 | 93 | 10.076 | 91 | 3.976 | 91 | 2.766 | 91 | 0.963 | 100 | 8.590 | 100 | 4.156 | 87.5 |
| Alcohol RT 21,5 (2-Undecanol/2-Dodecanol/2-Decanol) | 8.451 | 7 | 0.000 | 0 | 0.052 | 20 | 0.025 | 9 | 0.000 | 0 | 0.012 | 9 | 0.000 | 0 | 0.000 | 0 | 0.000 | 0 |
| Aldehyde RT 21,5 (Tridecanal) | 0.124 | 93 | 1.161 | 87 | 1.200 | 93 | 0.283 | 27 | 0.218 | 27 | 0.335 | 36 | 0.989 | 100 | 0.813 | 100 | 0.788 | 100 |
| Keton RT 21,5 (2-Decanone/2-Tridecanone) | 0.000 | 33 | 1.543 | 80 | 0.457 | 53 | 0.087 | 55 | 1.292 | 73 | 0.302 | 36 | 0.113 | 62.5 | 0.809 | 100 | 0.395 | 75 |
| 2-Buten-1-one, 1-(2,6,6-trimethyl-1,3-cyclohexadien-1-yl)-, (E)- | 0.000 | 0 | 0.000 | 0 | 0.009 | 7 | 0.000 | 0 | 0.000 | 0 | 0.000 | 0 | 0.000 | 0 | 0.000 | 0 | 0.000 | 0 |
| Aldehyde RT 21,6 | 8.833 | 0 | 0.000 | 0 | 0.000 | 0 | 0.660 | 64 | 0.641 | 64 | 0.797 | 64 | 0.000 | 0 | 0.000 | 0 | 0.000 | 0 |
| Benzaldehyde, 3,5-dimethyl- | 0.003 | 100 | 1.662 | 100 | 1.580 | 100 | 1.343 | 100 | 2.128 | 100 | 1.722 | 100 | 0.940 | 100 | 1.285 | 100 | 1.620 | 100 |
| Naphthalene, 1,2-dihydro-1,1,6-trimethyl- | 0.000 | 7 | 0.000 | 0 | 0.006 | 13 | 0.000 | 0 | 0.000 | 0 | 0.000 | 0 | 0.000 | 0 | 0.000 | 0 | 0.000 | 0 |
| Unknown alcohol RT 21,79 | 0.000 | 0 | 0.030 | 7 | 0.000 | 0 | 0.000 | 0 | 0.060 | 9 | 0.000 | 0 | 0.000 | 0 | 0.000 | 0 | 0.000 | 0 |
| Benzene, 1-methoxy-4-(1-propenyl)- | 0.024 | 0 | 0.006 | 7 | 0.005 | 7 | 0.004 | 9 | 0.000 | 0 | 0.017 | 9 | 0.236 | 25 | 0.310 | 12.5 | 0.386 | 25 |
| 2-Butanone, 4-(2,6,6-trimethyl-1-cyclohexen-1-yl)- | 0.012 | 13 | 0.177 | 27 | 0.045 | 40 | 0.015 | 9 | 0.042 | 27 | 0.014 | 9 | 0.061 | 25 | 0.043 | 37.5 | 0.015 | 25 |
| 1-Octanol, 2-Butyl-/1-Decanol, 2-ethyl- | 21.257 | 7 | 0.000 | 0 | 0.000 | 0 | 0.000 | 0 | 0.000 | 0 | 0.000 | 0 | 0.000 | 0 | 0.000 | 0 | 0.000 | 0 |
| Hexanoic acid | 0.016 | 100 | 25.099 | 100 | 44.927 | 100 | 59.549 | 100 | 31.140 | 100 | 65.593 | 100 | 86.939 | 100 | 68.278 | 100 | 159.059 | 100 |
| Naphthalene, 2-methyl- | 0.014 | 7 | 0.000 | 0 | 0.000 | 0 | 0.000 | 0 | 0.000 | 0 | 0.000 | 0 | 0.000 | 0 | 0.000 | 0 | 0.000 | 0 |
| Benzenemethanol, .alpha.,.alpha.,4-trimethyl- | 0.000 | 27 | 0.030 | 33 | 0.040 | 33 | 0.000 | 0 | 0.000 | 0 | 0.000 | 0 | 0.009 | 12.5 | 0.003 | 12.5 | 0.027 | 37.5 |
| Ethanone, 1-(2,4,6-trimethylphenyl)- | 7.974 | 0 | 0.000 | 0 | 0.000 | 0 | 0.000 | 0 | 0.000 | 0 | 0.000 | 0 | 0.000 | 0 | 0.000 | 0 | 0.000 | 0 |
| 5,9-Undecadien-2-one, 6,10-dimethyl- | 0.000 | 80 | 0.669 | 80 | 0.409 | 87 | 0.203 | 64 | 0.331 | 64 | 0.888 | 91 | 0.624 | 75 | 0.267 | 87.5 | 0.390 | 100 |
| 1-Tridecanol/1-Undecanol | 0.000 | 0 | 0.000 | 0 | 0.076 | 13 | 0.070 | 9 | 0.061 | 9 | 0.012 | 9 | 0.000 | 0 | 0.000 | 0 | 0.010 | 12.5 |
| Cycloalka/ene RT 22,1 | 0.000 | 0 | 0.000 | 0 | 0.000 | 0 | 0.000 | 0 | 0.040 | 9 | 0.000 | 0 | 0.000 | 0 | 0.000 | 0 | 0.000 | 0 |
| Keton RT 22,1 (2-Tetradecanone) | 0.000 | 0 | 0.174 | 27 | 0.022 | 7 | 0.000 | 0 | 0.046 | 9 | 0.019 | 9 | 0.000 | 0 | 0.060 | 25 | 0.000 | 0 |
| Aldehyde/alkene RT 22,2 | 0.167 | 0 | 0.054 | 7 | 0.023 | 7 | 0.000 | 0 | 0.014 | 9 | 0.016 | 9 | 0.000 | 0 | 0.000 | 0 | 0.000 | 0 |
| Propanoic acid, 2-methyl-,  3-hydroxy-2,4,4-trimethylpentyl ester | 0.000 | 33 | 0.084 | 13 | 0.183 | 40 | 0.097 | 45 | 0.070 | 27 | 0.066 | 27 | 0.014 | 25 | 0.052 | 25 | 0.036 | 25 |
| Benzyl alcohol | 14.187 | 0 | 0.006 | 7 | 0.007 | 13 | 0.000 | 0 | 0.000 | 0 | 0.000 | 0 | 0.005 | 12.5 | 0.000 | 0 | 0.011 | 12.5 |
| Aldehyde RT 22,3 (Tetradecanal) | 0.000 | 93 | 9.284 | 100 | 9.256 | 93 | 10.591 | 100 | 14.244 | 91 | 12.196 | 91 | 15.138 | 100 | 14.692 | 100 | 13.612 | 100 |
| Keton RT 22,3 (2-Dodecanone) | 0.000 | 0 | 0.336 | 27 | 0.062 | 13 | 0.000 | 0 | 0.197 | 9 | 0.064 | 9 | 0.000 | 0 | 0.194 | 12.5 | 0.000 | 0 |
| Benzenepropanoic acid, ethyl ester | 7.853 | 0 | 0.000 | 0 | 0.133 | 67 | 0.000 | 0 | 0.000 | 0 | 0.034 | 45 | 0.000 | 0 | 0.000 | 0 | 0.061 | 50 |
| Hexanoic acid, branched (4-Methylhexanoic acid) | 28.156 | 33 | 0.176 | 27 | 0.167 | 27 | 0.035 | 36 | 0.044 | 27 | 0.070 | 36 | 0.124 | 25 | 0.207 | 50 | 0.206 | 50 |
| Aldehyde RT 22,9 | 0.000 | 100 | 32.632 | 100 | 29.930 | 100 | 21.858 | 100 | 29.629 | 100 | 27.258 | 100 | 23.677 | 100 | 26.703 | 100 | 28.808 | 100 |
| 1-Octadecyne | 0.000 | 0 | 0.130 | 7 | 0.000 | 0 | 0.000 | 0 | 0.000 | 0 | 0.000 | 0 | 0.000 | 0 | 0.000 | 0 | 0.000 | 0 |
| 4-(2,6,6-Trimethylcyclohexa-1,3-dienyl)but-3-en-2-one | 0.000 | 0 | 0.260 | 7 | 0.069 | 7 | 0.000 | 0 | 0.000 | 0 | 0.000 | 0 | 0.000 | 0 | 0.000 | 0 | 0.000 | 0 |
| Hexanoic acid, 2-ethyl- | 12.318 | 0 | 0.000 | 0 | 0.000 | 0 | 0.000 | 0 | 0.020 | 18 | 0.032 | 18 | 0.000 | 0 | 0.062 | 12.5 | 0.091 | 12.5 |
| Heptanoic acid | 9.059 | 60 | 5.516 | 47 | 7.577 | 67 | 10.004 | 82 | 5.458 | 73 | 11.850 | 91 | 25.074 | 87.5 | 9.068 | 75 | 31.075 | 100 |
| 1-Dodecanol | 0.146 | 80 | 0.693 | 80 | 1.972 | 93 | 4.666 | 73 | 10.614 | 82 | 3.292 | 91 | 0.378 | 87.5 | 0.467 | 87.5 | 1.309 | 87.5 |
| Aldehyde/alkene RT 23,3 | 0.000 | 40 | 0.097 | 27 | 0.220 | 47 | 0.311 | 27 | 0.365 | 27 | 0.369 | 27 | 0.042 | 12.5 | 0.019 | 12.5 | 0.052 | 12.5 |
| 2-Butene, 2,3-dimethyl- | 0.000 | 0 | 0.000 | 0 | 0.000 | 0 | 0.000 | 0 | 0.000 | 0 | 0.000 | 0 | 0.000 | 0 | 0.000 | 0 | 0.000 | 0 |
| Keton RT 23,4 (2-Hexanone, 4-methyl-) | 0.000 | 0 | 0.152 | 13 | 0.055 | 20 | 0.000 | 0 | 0.121 | 27 | 0.019 | 9 | 0.000 | 0 | 0.148 | 37.5 | 0.014 | 12.5 |
| Phenol, 2-methoxy-4-methyl- | 0.008 | 0 | 0.000 | 0 | 0.000 | 0 | 0.000 | 0 | 0.014 | 9 | 0.000 | 0 | 0.000 | 0 | 0.000 | 0 | 0.000 | 0 |
| Bicyclo[4.1.0]heptan-3-one, 7,7-dimethyl-4-methylene-, (1R)- | 0.000 | 7 | 0.008 | 7 | 0.000 | 0 | 0.009 | 9 | 0.025 | 9 | 0.000 | 0 | 0.000 | 0 | 0.000 | 0 | 0.000 | 0 |
| Hexanoic acid, 2-methyl- | 0.000 | 0 | 0.000 | 0 | 0.000 | 0 | 0.000 | 0 | 0.044 | 9 | 0.000 | 0 | 0.000 | 0 | 0.000 | 0 | 0.000 | 0 |
| Acetic acid, 3-methylphenyl ester | 7.948 | 0 | 0.000 | 0 | 0.000 | 0 | 0.000 | 0 | 0.000 | 0 | 0.000 | 0 | 0.000 | 0 | 0.000 | 0 | 0.000 | 0 |
| 4-Hydroxy-2-methylacetophenone | 10.293 | 7 | 0.000 | 0 | 0.011 | 7 | 0.000 | 0 | 0.000 | 0 | 0.000 | 0 | 0.000 | 0 | 0.000 | 0 | 0.000 | 0 |
| Aldehyde RT 23,6 | 23.110 | 100 | 3.440 | 93 | 3.926 | 100 | 3.942 | 91 | 5.496 | 100 | 5.256 | 91 | 3.055 | 100 | 3.650 | 100 | 4.650 | 100 |
| Aldehyde RT 23,7 | 0.265 | 100 | 22.870 | 93 | 26.705 | 100 | 24.452 | 91 | 34.159 | 82 | 32.773 | 82 | 28.476 | 100 | 30.384 | 100 | 36.419 | 100 |
| 2-Pentadecanone | 9.345 | 27 | 1.679 | 33 | 0.886 | 33 | 0.000 | 0 | 0.000 | 0 | 0.000 | 0 | 0.000 | 0 | 0.000 | 0 | 0.000 | 0 |
| Phenol | 0.055 | 60 | 7.570 | 93 | 1.575 | 73 | 0.162 | 64 | 6.151 | 100 | 0.352 | 55 | 0.128 | 50 | 5.998 | 87.5 | 0.399 | 75 |
| 4-(2,6,6-Trimethylcyclohexa-1,3-dienyl)but-3-en-2-one | 0.000 | 7 | 0.094 | 7 | 0.030 | 7 | 0.011 | 9 | 0.000 | 0 | 0.023 | 9 | 0.000 | 0 | 0.000 | 0 | 0.000 | 0 |
| Alcohol RT 23,9 | 0.119 | 0 | 0.083 | 7 | 0.106 | 7 | 0.006 | 9 | 0.000 | 0 | 0.000 | 0 | 0.000 | 0 | 0.042 | 12.5 | 0.000 | 0 |
| Keton RT 24,0 (2-Pentadecanone/2-Tetradecanone/2-Decanone) | 0.000 | 7 | 1.817 | 47 | 0.821 | 33 | 0.169 | 18 | 2.482 | 73 | 0.946 | 64 | 0.119 | 37.5 | 1.528 | 87.5 | 0.788 | 87.5 |
| Diphenyl ether | 13.725 | 0 | 0.000 | 0 | 0.000 | 0 | 0.000 | 0 | 0.000 | 0 | 0.000 | 0 | 0.000 | 0 | 0.000 | 0 | 0.000 | 0 |
| Aldehyde RT 24,1 | 0.000 | 100 | 8.948 | 93 | 9.611 | 100 | 5.848 | 100 | 6.602 | 100 | 7.650 | 100 | 8.353 | 100 | 7.490 | 100 | 9.425 | 100 |
| Alkeen RT 24,15 | 0.000 | 0 | 0.215 | 7 | 0.000 | 0 | 0.000 | 0 | 0.000 | 0 | 0.000 | 0 | 0.000 | 0 | 0.000 | 0 | 0.000 | 0 |
| Phenol, 4-ethyl-2-methoxy- | 0.000 | 0 | 0.015 | 13 | 0.003 | 7 | 0.000 | 0 | 0.000 | 0 | 0.000 | 0 | 0.000 | 0 | 0.000 | 0 | 0.000 | 0 |
| 2(3H)-Furanone, 5-ethyldihydro- | 0.000 | 0 | 0.000 | 0 | 0.007 | 7 | 0.000 | 0 | 0.000 | 0 | 0.000 | 0 | 0.000 | 0 | 0.000 | 0 | 0.000 | 0 |
| Alkeen RT 24,29 | 0.000 | 0 | 0.016 | 7 | 0.000 | 0 | 0.000 | 0 | 0.000 | 0 | 0.000 | 0 | 0.000 | 0 | 0.000 | 0 | 0.000 | 0 |
| Benzenepropanol | 8.915 | 0 | 0.000 | 0 | 0.009 | 13 | 0.000 | 0 | 0.000 | 0 | 0.000 | 0 | 0.000 | 0 | 0.000 | 0 | 0.000 | 0 |
| Octanoic Acid | 0.000 | 53 | 2.069 | 67 | 3.289 | 60 | 6.446 | 64 | 10.832 | 73 | 10.677 | 82 | 2.654 | 87.5 | 2.260 | 75 | 6.772 | 87.5 |
| 2-Propenal, 3-phenyl- | 8.274 | 0 | 0.000 | 0 | 0.000 | 0 | 0.013 | 18 | 0.010 | 9 | 0.011 | 9 | 0.000 | 0 | 0.000 | 0 | 0.000 | 0 |
| Caryophyllenyl alcohol | 0.063 | 67 | 0.155 | 60 | 0.204 | 53 | 0.134 | 27 | 0.194 | 18 | 0.093 | 36 | 0.297 | 75 | 0.265 | 62.5 | 0.299 | 62.5 |
| 1-Tridecanol/1-Undecanol | 0.000 | 13 | 0.072 | 7 | 0.000 | 0 | 0.063 | 9 | 0.079 | 9 | 0.078 | 9 | 0.101 | 12.5 | 0.014 | 12.5 | 0.076 | 12.5 |
| 1-Naphthalenol, decahydro-4a-methyl-8-methylene-2-(1-methylethyl)-, [1R-(1à,2á,4aá | 0.043 | 0 | 0.000 | 0 | 0.000 | 0 | 0.056 | 9 | 0.000 | 0 | 0.000 | 0 | 0.000 | 0 | 0.000 | 0 | 0.000 | 0 |
| 1-Penten-3-one, 1-(2,6,6-trimethyl-1-cyclohexen-1-yl)- | 0.093 | 7 | 0.000 | 0 | 0.000 | 0 | 0.000 | 0 | 0.000 | 0 | 0.000 | 0 | 0.017 | 12.5 | 0.000 | 0 | 0.000 | 0 |
| Keton RT 24,6 (2-Tetradecanone) | 0.000 | 7 | 0.863 | 20 | 0.582 | 20 | 0.000 | 0 | 0.187 | 9 | 0.109 | 18 | 0.000 | 0 | 0.145 | 12.5 | 0.057 | 12.5 |
| 2-Tetradecanone | 28.842 | 0 | 0.116 | 13 | 0.029 | 7 | 0.000 | 0 | 0.000 | 0 | 0.000 | 0 | 0.000 | 0 | 0.000 | 0 | 0.000 | 0 |
| Phenol, 4-methyl- | 8.262 | 100 | 29.659 | 93 | 29.752 | 100 | 21.287 | 100 | 30.506 | 100 | 35.400 | 100 | 12.934 | 75 | 16.324 | 87.5 | 19.946 | 87.5 |
| Aldehyde RT 24,7 | 9.280 | 13 | 0.941 | 27 | 1.268 | 20 | 0.651 | 27 | 0.554 | 18 | 0.000 | 0 | 3.056 | 25 | 0.414 | 12.5 | 0.418 | 12.5 |
| Cyclohexanecarboxylic acid | 0.000 | 33 | 1.536 | 33 | 1.481 | 27 | 0.538 | 18 | 0.000 | 0 | 0.242 | 9 | 3.003 | 50 | 2.980 | 50 | 2.453 | 50 |
| unknown 165/177/180/192 | 0.000 | 0 | 0.006 | 7 | 0.010 | 7 | 0.000 | 0 | 0.000 | 0 | 0.000 | 0 | 0.000 | 0 | 0.000 | 0 | 0.000 | 0 |
| Keton RT 24,8 | 0.040 | 0 | 0.000 | 0 | 0.000 | 0 | 0.000 | 0 | 0.000 | 0 | 0.000 | 0 | 0.000 | 0 | 0.114 | 12.5 | 0.000 | 0 |
| Phenol, 2-methyl-5-(1-methylethyl)- | 0.002 | 20 | 0.052 | 20 | 0.039 | 20 | 0.000 | 0 | 0.000 | 0 | 0.000 | 0 | 0.000 | 0 | 0.000 | 0 | 0.000 | 0 |
| unknown 133/159/177 | 0.023 | 7 | 0.000 | 0 | 0.000 | 0 | 0.000 | 0 | 0.000 | 0 | 0.000 | 0 | 0.000 | 0 | 0.000 | 0 | 0.004 | 12.5 |
| Alcohol RT 25,0 | 0.000 | 13 | 0.072 | 20 | 0.276 | 40 | 0.065 | 9 | 0.354 | 18 | 0.215 | 45 | 0.015 | 12.5 | 0.130 | 50 | 0.353 | 62.5 |
| Keton RT 25,1 (2-Hexanone, 4-methyl-) | 0.037 | 0 | 0.000 | 0 | 0.000 | 0 | 0.000 | 0 | 0.000 | 0 | 0.000 | 0 | 0.000 | 0 | 0.025 | 12.5 | 0.000 | 0 |
| 2-Pentadecanone, 6,10,14-trimethyl- | 0.000 | 7 | 0.000 | 0 | 0.000 | 0 | 0.000 | 0 | 0.000 | 0 | 0.000 | 0 | 0.000 | 0 | 0.000 | 0 | 0.000 | 0 |
| 2-Nonadecanone | 0.000 | 0 | 0.066 | 7 | 0.000 | 0 | 0.000 | 0 | 0.000 | 0 | 0.000 | 0 | 0.000 | 0 | 0.000 | 0 | 0.000 | 0 |
| 3-Buten-2-one, 1-(2,3,6-trimethylphenyl)- | 16.386 | 0 | 0.000 | 0 | 0.000 | 0 | 0.000 | 0 | 0.000 | 0 | 0.000 | 0 | 0.000 | 0 | 0.000 | 0 | 0.000 | 0 |
| Aldehyde RT 25,2 | 0.007 | 93 | 18.172 | 87 | 22.298 | 93 | 7.246 | 100 | 9.564 | 91 | 14.537 | 100 | 13.113 | 100 | 15.060 | 100 | 21.779 | 100 |
| Ethanol, 2-phenoxy- | 1.015 | 7 | 0.000 | 0 | 0.000 | 0 | 0.000 | 0 | 0.000 | 0 | 0.000 | 0 | 0.000 | 0 | 0.000 | 0 | 0.000 | 0 |
| Aldehyde/alkene RT 25,5 | 0.381 | 67 | 1.550 | 67 | 1.598 | 67 | 0.279 | 27 | 0.297 | 18 | 0.705 | 55 | 0.494 | 37.5 | 0.247 | 50 | 0.656 | 75 |
| Nonanoic acid | 0.359 | 20 | 0.646 | 13 | 0.278 | 13 | 0.000 | 0 | 0.394 | 18 | 0.009 | 9 | 0.147 | 25 | 0.072 | 12.5 | 0.000 | 0 |
| Alcohol RT 25,6 | 0.067 | 13 | 0.802 | 27 | 0.346 | 20 | 0.210 | 18 | 0.593 | 27 | 0.594 | 36 | 0.348 | 25 | 0.075 | 12.5 | 0.415 | 37.5 |
| Phenol, 4-ethyl- | 8.925 | 27 | 0.057 | 20 | 0.059 | 27 | 0.512 | 45 | 0.383 | 36 | 0.695 | 45 | 0.745 | 50 | 0.714 | 75 | 0.537 | 50 |
| Benzoic acid, 4-ethoxy-, ethyl ester | 0.000 | 73 | 0.326 | 80 | 0.348 | 80 | 0.524 | 64 | 0.329 | 73 | 0.381 | 73 | 0.223 | 75 | 0.371 | 87.5 | 0.309 | 75 |
| Cyclohexanemethanol | 0.000 | 0 | 0.000 | 0 | 0.000 | 0 | 0.000 | 0 | 0.000 | 0 | 0.000 | 0 | 0.000 | 0 | 0.000 | 0 | 0.000 | 0 |
| Unknown RT 25,8 | 0.024 | 0 | 0.000 | 0 | 0.000 | 0 | 0.000 | 0 | 0.000 | 0 | 0.000 | 0 | 0.000 | 0 | 0.000 | 0 | 0.000 | 0 |
| Alkaan RT 25.82 | 0.148 | 7 | 0.002 | 7 | 0.000 | 0 | 0.000 | 0 | 0.000 | 0 | 0.000 | 0 | 0.000 | 0 | 0.000 | 0 | 0.000 | 0 |
| Phenol, 2-methyl-5-(1-methylethyl)- | 0.072 | 27 | 0.085 | 27 | 0.047 | 13 | 0.020 | 27 | 0.016 | 27 | 0.031 | 27 | 0.077 | 50 | 0.030 | 37.5 | 0.016 | 25 |
| 1-Tridecanol | 0.000 | 7 | 0.043 | 13 | 0.076 | 13 | 0.000 | 0 | 0.000 | 0 | 0.000 | 0 | 0.000 | 0 | 0.000 | 0 | 0.000 | 0 |
| Alcohol RT 26,2 | 0.000 | 0 | 0.027 | 7 | 0.154 | 20 | 0.000 | 0 | 0.000 | 0 | 0.000 | 0 | 0.000 | 0 | 0.000 | 0 | 0.000 | 0 |
| n-Decanoic acid | 0.860 | 0 | 0.000 | 0 | 0.000 | 0 | 0.291 | 9 | 0.000 | 0 | 0.000 | 0 | 0.000 | 0 | 0.000 | 0 | 0.000 | 0 |
| Alcohol RT 26,5 (Tridecanol) | 8.610 | 33 | 2.267 | 53 | 2.676 | 47 | 2.727 | 55 | 6.554 | 82 | 7.817 | 82 | 0.684 | 62.5 | 0.297 | 12.5 | 1.038 | 37.5 |
| 7-Hexadecenal, (Z)- | 0.003 | 7 | 0.000 | 0 | 0.000 | 0 | 0.000 | 0 | 0.000 | 0 | 0.000 | 0 | 0.000 | 0 | 0.000 | 0 | 0.256 | 12.5 |
| Phenol, 4-propyl- | 0.261 | 7 | 0.006 | 7 | 0.000 | 0 | 0.006 | 9 | 0.005 | 9 | 0.006 | 9 | 0.000 | 0 | 0.000 | 0 | 0.000 | 0 |
| Alkaan RT 26.8 | 13.442 | 7 | 0.000 | 0 | 0.000 | 0 | 0.000 | 0 | 0.000 | 0 | 0.000 | 0 | 0.000 | 0 | 0.000 | 0 | 0.000 | 0 |
| 3,4-Dimethyl-2-(3-methyl-butyryl)-benzoic acid, methyl ester | 0.000 | 100 | 7.205 | 100 | 5.972 | 100 | 3.318 | 100 | 4.540 | 100 | 5.559 | 100 | 4.233 | 100 | 5.192 | 100 | 4.408 | 100 |
| Phenol, 2,4-bis(1,1-dimethylethyl)- | 0.000 | 0 | 0.000 | 0 | 0.000 | 0 | 0.000 | 0 | 0.000 | 0 | 0.000 | 0 | 0.000 | 0 | 0.000 | 0 | 0.000 | 0 |
| 1-Hexadecanol | 10.194 | 0 | 0.167 | 13 | 0.000 | 0 | 0.000 | 0 | 0.000 | 0 | 0.000 | 0 | 0.000 | 0 | 0.000 | 0 | 0.000 | 0 |
| Diphenyl sulfide | 0.083 | 100 | 0.751 | 100 | 0.592 | 100 | 0.465 | 100 | 0.603 | 100 | 0.513 | 100 | 0.381 | 100 | 0.412 | 100 | 0.414 | 100 |
| 1-Hexadecanol | 0.000 | 7 | 0.016 | 7 | 0.033 | 7 | 0.000 | 0 | 0.000 | 0 | 0.030 | 9 | 0.103 | 12.5 | 0.798 | 12.5 | 0.000 | 0 |
| 1,2-Benzenedicarboxylic acid, diheptyl ester | 0.000 | 0 | 0.000 | 0 | 0.000 | 0 | 0.000 | 0 | 0.007 | 9 | 0.000 | 0 | 0.000 | 0 | 0.000 | 0 | 0.000 | 0 |
| Aldehyde/alkene RT 27,6 | 0.126 | 0 | 0.139 | 13 | 0.197 | 13 | 0.000 | 0 | 0.000 | 0 | 0.000 | 0 | 0.000 | 0 | 0.000 | 0 | 0.008 | 12.5 |
| 2(3H)-Furanone, 5-heptyldihydro- | 0.000 | 33 | 0.422 | 47 | 0.428 | 60 | 0.190 | 55 | 0.565 | 73 | 0.676 | 82 | 0.309 | 62.5 | 1.079 | 62.5 | 0.585 | 87.5 |
| Undecanoic acid | 0.000 | 0 | 0.000 | 0 | 0.000 | 0 | 0.000 | 0 | 0.000 | 0 | 0.000 | 0 | 0.000 | 0 | 0.031 | 12.5 | 0.022 | 12.5 |
| Benzene, 1,4-dimethyl-2,5-bis(1-methylethyl)- | 9.240 | 0 | 0.004 | 7 | 0.000 | 0 | 0.000 | 0 | 0.000 | 0 | 0.000 | 0 | 0.000 | 0 | 0.000 | 0 | 0.000 | 0 |
| Indole | 0.000 | 73 | 2.357 | 87 | 0.370 | 60 | 0.188 | 64 | 3.274 | 100 | 2.939 | 73 | 0.158 | 62.5 | 1.444 | 87.5 | 0.094 | 62.5 |
| 5H-1-Pyrindine | 0.083 | 0 | 0.042 | 7 | 0.000 | 0 | 0.000 | 0 | 0.000 | 0 | 0.000 | 0 | 0.000 | 0 | 0.000 | 0 | 0.000 | 0 |
| Dodecanoic acid | 0.047 | 7 | 0.000 | 0 | 0.000 | 0 | 0.140 | 9 | 1.003 | 9 | 2.990 | 9 | 0.000 | 0 | 0.027 | 12.5 | 0.000 | 0 |
| Alkaan RT 28.70 | 9.850 | 7 | 0.000 | 0 | 0.000 | 0 | 0.000 | 0 | 0.000 | 0 | 0.000 | 0 | 0.000 | 0 | 0.000 | 0 | 0.000 | 0 |
| 1H-Indole, 3-methyl- | 0.028 | 60 | 2.140 | 73 | 1.364 | 67 | 1.157 | 45 | 2.545 | 64 | 1.550 | 64 | 0.811 | 75 | 1.753 | 100 | 1.188 | 87.5 |
| 3,4-Dimethyl-2-(3-methyl-butyryl)-benzoic acid, methyl ester | 10.063 | 7 | 0.000 | 0 | 0.000 | 0 | 0.002 | 9 | 0.000 | 0 | 0.000 | 0 | 0.000 | 0 | 0.000 | 0 | 0.000 | 0 |
| 1,2-Benzenedicarboxylic acid, diheptyl ester | 0.005 | 100 | 0.332 | 87 | 0.176 | 87 | 0.425 | 100 | 0.101 | 55 | 0.112 | 82 | 0.351 | 87.5 | 0.078 | 62.5 | 0.118 | 62.5 |
| Benzeneacetic acid | 0.000 | 7 | 0.024 | 7 | 0.006 | 7 | 0.005 | 9 | 0.000 | 0 | 0.002 | 9 | 0.000 | 0 | 0.000 | 0 | 0.003 | 12.5 |
| 3-Phenyl-propionic acid, isopropyl ester | 0.000 | 0 | 0.000 | 0 | 0.000 | 0 | 0.000 | 0 | 0.000 | 0 | 0.000 | 0 | 0.000 | 0 | 0.000 | 0 | 0.000 | 0 |
| Benzenepropanoic acid | 0 | 0 | 0.000 | 0 | 0.000 | 0 | 0.000 | 0 | 0.000 | 0 | 0.000 | 0 | 0.000 | 0 | 0.000 | 0 | 0.000 | 0 |

© 2015 by the authors; licensee MDPI, Basel, Switzerland. This article is an open access article distributed under the terms and conditions of the Creative Commons Attribution license (http://creativecommons.org/licenses/by/4.0/).
